# Assessment of a home-based standing frame programme in people with progressive multiple sclerosis (SUMS): a pragmatic, multi-centre, randomised, controlled trial and cost-effectiveness analysis

**DOI:** 10.1016/S1474-4422(19)30190-5

**Published:** 2019-08

**Authors:** Jennifer Freeman, Wendy Hendrie, Louise Jarrett, Annie Hawton, Andrew Barton, Rachel Dennett, Ben Jones, John Zajicek, Siobhan Creanor

**Affiliations:** a Faculty of Health and Human Sciences, School of Health Professions, University of Plymouth, Peninsula Allied Health Centre, Plymouth, UK; b Norwich MS Centre, Norwich, UK; c Mardon Neurorehabilitation Centre, Royal Devon and Exeter NHS Foundation Trust, Exeter, UK; d University of Exeter Medical School, Health Economics Group, University of Exeter, Exeter, UK; e NIHR Research Design Service, Faculty of Medicine and Dentistry, University of Plymouth, Plymouth, UK; f Medical Statistics Group, Faculty of Medicine and Dentistry, University of Plymouth, Plymouth, UK; g Peninsula Clinical Trials Unit, Faculty of Medicine and Dentistry, University of Plymouth, Plymouth, UK; h School of Medicine, Medical and Biological Sciences, University of St Andrews, St Andrews, UK

## Abstract

**Background:**

People severely impaired with progressive multiple sclerosis spend much of their day sitting, with very few options to improve motor function. As a result, secondary physical and psychosocial complications can occur. Effective and feasible self-management strategies are needed to reduce sedentary behaviour and enhance motor function. In this study, we aimed to assess the clinical and cost effectiveness of a home-based, self-managed, standing frame programme.

**Methods:**

SUMS was a pragmatic, multicentre, randomised controlled superiority trial of people with progressive multiple sclerosis and severe mobility impairment, undertaken in eight centres from two regions in the UK. The study had assessor-blinded outcome assessments with use of clinician-rated and patient-rated measures at baseline, 20 weeks, and 36 weeks. After baseline assessment, participants were randomised (1:1) by computer-generated assignment to either a standing frame programme plus usual care or usual care alone. The intervention consisted of two home-based physiotherapy sessions (60 min each) to set up the standing frame programme, supported by six follow-up telephone calls (15 min per call). Participants were asked to stand for 30 min, three times per week over 20 weeks, and encouraged to continue in the longer term, although no further physiotherapy support was provided. The primary clinical outcome was motor function measured by the Amended Motor Club Assessment (AMCA) score at week 36, analysed in the modified intention-to-treat population (excluding only patients who were deemed ineligible after randomisation, those who withdrew from the trial and were unwilling for their previously collected data to be used, or those who did not provide baseline and week 36 measurements). A 9-point AMCA score change was considered clinically meaningful a priori. Adverse events were collected through a daily preformatted patient diary throughout the 36 weeks and analysed in the modified intention-to-treat population. An economic assessment established the resources required to provide the standing frame programme, estimated intervention costs, and estimate cost effectiveness. This trial is registered with the International Standard Randomised Controlled Trials, number ISRCTN69614598.

**Findings:**

Between Sept 16, 2015, and April 28, 2017, 285 people with progressive multiple sclerosis were screened for eligibility, and 140 were randomly assigned to either the standing frame group (n=71) or the usual care group (n=69). Of these, 122 completed the primary outcome assessment (61 participants in both groups) for the modified intention-to-treat analysis. The use of the standing frame resulted in a significant increase in AMCA score compared with that for usual care alone, with a fully adjusted between-group difference in AMCA score at 36 weeks of 4·7 points (95% CI 1·9–7·5; p=0·0014). For adverse events collected through patient diaries, we observed a disparity between the two groups in the frequency of short-term musculoskeletal pain (486 [41%] of 1188 adverse events in the standing frame group *vs* 160 [22%] of 736 adverse events in the usual care group), which was potentially related to the intervention. The musculoskeletal pain lasted longer than 7 days in five participants (two in the standing frame group and three in the usual care group). No serious adverse events related to the study occurred. The standing frame group had a mean 0·018 (95% CI −0·014 to 0·051) additional quality-adjusted life-years (QALYs) compared with those of the usual care group, and the estimated incremental cost-per-QALY was approximately £14 700.

**Interpretation:**

The standing frame programme significantly increased motor function in people with severe progressive multiple sclerosis, although not to the degree that was considered a priori as clinically meaningful. The standing frame is one of the first physiotherapy interventions to be effective in this population. We suggest that the programme is feasible as a home-based, self-managed intervention that could be routinely implemented in clinical practice in the UK.

**Funding:**

UK National Institute of Health Research.

**Research in context**

**Evidence before this study**

We searched electronic databases (MEDLINE, AMED, CINAHL, Embase, PsycINFO, and PEDro) for manuscripts published in English and with study populations aged older than 18 years, from database inception to Aug 1, 2018. Search terms were “multiple sclerosis” and “standing frames”, “standing tables”, or “standing wheelchairs”. We also checked the reference lists from identified papers and searched ClinicalTrials.gov and the International Standard Randomised Controlled Trials registry. No adequately powered randomised controlled trials assessing the clinical or cost effectiveness of a standing intervention were identified. Our search revealed one systematic review of standing in people with upper motor neuron disorders that cited a small pilot randomised trial in people with multiple sclerosis (n=6) and one mixed-methods study (AB case study design plus interviews, n=9), neither of which exclusively recruited people with progressive multiple sclerosis. To our knowledge, no randomised controlled trials of standing frame use in people with multiple sclerosis have been undertaken since our literature search.

**Added value of this study**

To our knowledge, the SUMS study is the largest randomised controlled trial assessing physical rehabilitation in people with progressive multiple sclerosis. It is the first assessor-blinded, multicentre, randomised trial to investigate the clinical and cost effectiveness, safety, and tolerability of a supported standing frame programme plus usual care versus usual care alone in people with progressive multiple sclerosis whose standing balance and walking is severely impaired. The standing frame programme was well tolerated in people with multiple sclerosis who were unable to walk or whose mobility was limited to a maximum of 20 m with a bilateral walking aid. The standing programme significantly increased motor function in people with progressive multiple sclerosis, although not to the degree that was considered a priori as clinically meaningful. The response of participants varied regarding standing but, on average, longer standing times were associated with significantly greater improvements in motor function, with the confidence intervals containing the a priori clinically meaningful improvement. Our cost-effectiveness analysis showed that the standing frame programme had an estimated incremental cost of approximately £14 700 per quality-adjusted life-year (QALY) and a 0·52 to 0·61 probability of being cost effective at the National Institute of Health and Care Excellence threshold of £20 000–30 000 per QALY.

**Implications of all the available evidence**

The use of a home-based, self-managed standing frame programme could improve motor function in individuals with progressive multiple sclerosis. Our study is an important addition to the evidence-base for supported standing, for which high-level evidence is currently lacking.

## Introduction

Multiple sclerosis is a progressive, neurological condition that affects 2·5 million people worldwide. The disease impacts all aspects of patients' lives, having substantial and adverse effects on quality of life. Multiple sclerosis is associated with high direct and indirect costs to patients, their families, and society. These costs are highly correlated with increasing immobility.^1^

Mobility is a major concern for people with multiple sclerosis.^2^ It is estimated that, within 10–15 years of diagnosis, approximately 80% of people will have impaired mobility. Eventually, an estimated 25% of patients are wheelchair dependent.^3^ Mobility spans more than walking, including also standing, transferring, and moving in bed.^4^ These are important activities for maintaining independence, particularly for people who are severely physically impaired. Individuals with progressive multiple sclerosis spend much of their day sitting,^5^ often with reduced ability to change position. In response, insidious but preventable secondary complications can occur, including muscle wasting, reduced skin integrity, spasms, constipation, depression, and lowered self-esteem.^6^ These problems can compound the primary neurological disability, accelerating loss of independence, and can even be mistaken for disease progression. Furthermore, long periods of sitting time are associated with increased risks of morbidity and mortality.^5^ The clinical importance of these issues is underlined by their consistent prominence in policy documents for long-term neurological conditions.^4^, ^7^

Strong evidence exists that increases in physical activity can improve mobility and minimise secondary health problems in people with mild to moderate multiple sclerosis,^8^ and evidence suggests that this might also be the case for people with severe multiple sclerosis.^9^, ^10^ Despite this evidence, up to 78% of people with multiple sclerosis do not participate in meaningful physical activity.^11^ There can be considerable barriers to keeping active when mobility impairment is severe.^12^ Interventions have typically been resource intensive, entailing regular supervised sessions by a physiotherapist or sports therapist, in an outpatient or hospital setting, and relying on expensive equipment that cannot be used in the home environment.^9^, ^10^ Moreover, more data are needed regarding adherence when supervision ceases.

Finite health-care resources mean that ongoing supervision of physical activity programmes is rarely possible. Effective self-management strategies, which are low cost and realistic to implement, are needed for people with severe physical limitations to optimise their engagement in physical activity. Regular supported standing with use of standing frames, which can be used within people's homes, is one such option. Standing frames enable individuals with restricted mobility, balance, or lower limb or trunk control the opportunity to spend time in supported standing. Proposed benefits of standing include strengthening antigravity muscles, providing prolonged weight-bearing muscle stretch, enhancing respiratory function, and maintaining bone density.^6^ Although preliminary evidence has shown benefit for their use in people with multiple sclerosis,^13^, ^14^, ^15^ no appropriately powered randomised controlled trials have been done. In line with the conclusions of a systematic review^6^ that such evidence was needed, we aimed to assess whether a home-based standing frame programme was clinically effective and to explore its cost-effectiveness in people with severe, progressive multiple sclerosis.

## Methods

### Study design and participants

The trial methods, previously published in detail,^16^ are briefly described in line with existing guidelines.^17^, ^18^, ^19^, ^20^ The SUMS study was an individually randomised, controlled, pragmatic, multi-centre, superiority trial with masked outcome assessments in people with progressive multiple sclerosis. Participants were randomly assigned to receive either usual care or usual care plus a standing programme, with masked assessments done at baseline, 20 weeks post-randomisation (aligned with the end of the protocol intervention period for those allocated to the intervention group), and again 16 weeks afterwards (36 weeks post-randomisation).

Participants were recruited through eight health-care organisations, including the UK National Health Service (NHS) Trusts, social enterprises, and third sector multiple sclerosis therapy centres, in two regions (Devon–Cornwall and East Anglia) of the UK. Individuals were invited consecutively until the allocated number of standing frames (dependent on commissioning costs) at each health-care organisation had been reached. Key inclusion criteria were age older than 18 years, a diagnosis of progressive multiple sclerosis (primary or secondary) according to McDonald's criteria,^21^ and a score of 6·5–8·0 on the Expanded Disability Status Scale (EDSS). Key exclusion criteria were being within 3 months of ceasing a multiple sclerosis disease-modifying drug, receiving steroid treatment within the preceding month, or participating in another clinical trial. Full inclusion and exclusion criteria are reported in the protocol paper.^16^

This study was ethically approved by the NHS Health Research Authority Committee South West—Frenchay Research Ethics Committee (15/SW/0088). Participants provided written informed consent before enrolment or undertaking any study-related procedures.

People with multiple sclerosis were actively involved throughout the study, including in the development of the research questions, study design, trial management and steering groups, writing of study materials, and dissemination activities.

### Randomisation and masking

The 1:1 allocation sequence was done with random-sized permuted blocks, stratified by region (Devon–Cornwall or East Anglia) and baseline EDSS score (≤7·0 or ≥7·5). The sequence was computer generated in conjunction with an independent statistician who had no further involvement in the trial. The randomisation list and the programme that generated it were stored in a secure network location within the Peninsula Clinical Trials Unit, registered with the UK Clinical Research Collaboration, accessible only to those responsible for providing the system. Participants were randomly assigned after baseline assessment, with the masked assessor inputting the participant details directly into the randomisation website.

It was not possible to mask trial participants, carers, or treating physiotherapists because of the nature of the intervention. However, outcome assessors (research therapists) were masked to treatment allocation, and all assessments were done independently and away from the participant's home. At each assessment timepoint, research therapists were asked whether they were unmasked to group allocation; 114 (89%) of 128 answers at week 20 and 110 (87%) of 126 at week 36 were “no”. The trial statisticians were masked for the primary analysis of the primary outcome.

### Procedures

Participants allocated to the standing frame group were issued with a wooden Oswestry standing frame (Theo Davies & Sons, Wrexham, UK), funded through the UK NHS commissioning process and delivered to the participant's home before the first physiotherapy session. The person with multiple sclerosis and their standing assistant (typically their spouse) engaged in two face-to-face, home-based, 60-min physiotherapy sessions, aimed at setting up, implementing, and progressing the standing programme according to ability, supplemented by online advice and DVDs. These were supported by six scripted telephone calls (15 min per call) that used a behaviour-change approach^22^ to increase the participant's self-efficacy, intended to enhance long-term engagement.

In line with previous research,^14^ participants were asked to stand in the frame for 30 min three times per week over 20 weeks, and to record the frequency and duration of each stand in a daily diary. This allowed for a graduated introduction to standing. At the end of the 20-week period, participants were encouraged to continue to regularly stand, although no further physiotherapy support was provided. On trial completion, participants were able to keep the frame, providing they used it at least once per week.

The use of standing frames is a recognised core skill for UK-trained neurological physiotherapists. To standardise and optimise implementation of the intervention, we provided educational materials and assessed fidelity to them.^16^ All participants received their usual health and social service input throughout the study period.^16^ This input was recorded on a self-report health-care and social-care resource form, which included changes in medication.

### Outcomes

Validated outcome measures included clinician-rated assessments and self-reported questionnaires. The primary outcome was motor function as measured by the Amended Motor Club Assessment (AMCA) score^23^ at the primary endpoint of 36 weeks post-randomisation. This score was developed for use by physiotherapists in a clinical setting to assess motor function in people with multiple sclerosis and has shown validity, reliability, and responsiveness.^14^, ^23^, ^24^ The AMCA score (range 0–76) is the sum of two subscores. The functional activity subscore (16 items, each scored 0–3) comprises key functional activities of the trunk and lower limbs, such as rolling in bed, sit-to-stand, and sitting and standing balance. The lower limb movement subscore (14 items, each scored 0–2) rates motor impairment by grading hip and knee flexion and knee flexion and dorsiflexion in lying, sitting, and standing positions.

The secondary outcomes, at weeks 20 and 36, were measurements of explanatory physical impairments (length of hip flexors, hamstrings and ankle plantar flexors [manual goniometry], knee extensor strength [hand-held dynamometer], spasm frequency [Penn Spasm Frequency Scale], and forced expiratory volume at 1 s [hand-held spirometer]);^16^ clinical outcomes (bowel and bladder control [bladder and bowel control scales], sitting balance [modified functional reach in sitting], and falls frequency); and quality of life (29-item Multiple Sclerosis Impact Scale [MSIS-29, version 2]). AMCA score at week 20 and the two AMCA subscores at week 36 were also measured as secondary outcomes. Participants were classified as fallers if they self-reported falling on 2 or more days during three different periods: up to week 20, up to week 36, and between weeks 21 and 36.

All participants were asked to record new symptoms, falls, and medication changes in a daily preformatted diary. Intervention participants were asked to record frequency and duration of standing sessions and any adverse events they had. A serious adverse event was defined as an untoward occurrence that resulted in death, was life-threatening, required hospital admission, or was considered medically significant by the investigator. An embedded qualitative component explored the contemporaneous subjective experiences of using a standing frame within daily life through audio-recorded diaries by a subgroup of intervention participants. These data will be reported in a future publication.

### Statistical analysis

The target sample size was based on comparing AMCA scores at week 36 between allocated groups, adjusting for baseline AMCA score, and detecting a minimal clinically important difference of 9 points, assuming an estimated SD 20 for AMCA score and estimated correlation of 0·55 between baseline and week 36 AMCA scores.^16^ The detection of a 9-point between-group difference with 80% power and at a 5% significance level required follow-up data from 55 participants per group. We allowed for 20% loss to follow-up or non-completion of primary outcome and set the recruitment target at 140 participants.

The analyses were pre-specified in a statistical analysis plan approved by the trial steering committee before analysis started, except for the analysis method for spasm frequency. Primary analyses were adjusted for the stratification factors (region and baseline EDSS) as fixed effects and baseline scores where appropriate (ie, fully adjusted models); results adjusted for baseline scores alone are also presented. Estimated between-group differences are presented with two-sided 95% CIs, with the two-sided significance level for hypothesis testing set at 5%. The analyses were done with Stata SE (version 14.2).

The primary analysis population was defined as all participants who completed baseline and 36-week assessments. The primary analysis of the primary outcome, AMCA score at 36 weeks, followed a modified intention-to-treat approach, regardless of compliance to the intervention, but did exclude patients who were deemed ineligible after randomisation, those who withdrew from the trial and were unwilling for their previously collected data to be used, or those who did not provide baseline and week 36 measurements (ie, there was no imputation of missing baseline or week 36 scores for the primary analysis), and used an analysis of covariance (ANCOVA) approach. As prespecified in the statistical analysis plan, Complier Average Causal Effect (CACE) sensitivity analyses were done on the 36-week AMCA scores. This method provides an unbiased estimate of the intervention effect, based on participants who complied with the standing intervention protocol.^25^ The agreed statistical analysis plan listed six compliance definitions that could trigger a CACE analysis^25^ (appendix p 2), if at least 20% of participants allocated to the intervention group were classed as non-compliers in the definition. The CACE analysis, triggered for all six definitions, used two-stage least squares instrumental variable regression, with treatment allocation as the instrument for the binary compliance variable and adjustment for baseline AMCA score, region, and EDSS category.^25^

A repeated-measures model was fitted to the post-baseline AMCA scores, including adjustment for baseline AMCA score, stratification variables, and the interaction term between allocated group and timepoint. Between-group pairwise comparisons at 20 and 36 weeks were calculated with use of marginal linear predictions and CIs from the fitted model.

All secondary outcomes were analysed on a modified intention-to-treat basis, with an ANCOVA approach, for both fully adjusted models and models with adjustment for baseline measures alone, except spasm frequency and falls. Ordinal logistic regression was prespecified for the analysis of the 5-level Penn Spasm Frequency Scale; however, because of insufficient numbers in some of the response categories, a dichotomisation of no spasms–mild spasms versus infrequent spasms–more than 1 per h–more than 10 per h was agreed. We used logistic regression to analyse the dichotomised Penn Spasm Frequency Scale and the binary outcome of fallers–non-fallers with adjustment for stratification factors.

We did a within-trial cost-effectiveness analysis. This estimated the additional costs of delivering the intervention, costs associated with health, social care, carer and patient resource use, and quality-adjusted life-years (QALYs) over the 36-week trial period. QALYs were estimated with use of self-report EQ-5D-5L (the five-level version of EQ-5D, a standardised generic instrument for measuring health status) data collected at baseline and at 20-week and 36-week follow-up, and by applying the so-called cross-walk algorithm^3^ to provide QALY weights from the UK general population valuation survey of the three-level version of EQ-5D.^4^ The primary perspective was the UK NHS and Personal Social Services (PSS), with a broader societal perspective considered in sensitivity analyses. Detailed methods are provided in the appendix (pp 3–9).

This trial is registered with the International Standard Randomised Controlled Trials, number ISRCTN69614598.

### Role of the funding source

This was an investigator-initiated study. The sponsor and funders of the study had no role in study design, data collection, data analysis, data interpretation, or writing of the report. All authors had full access to all the data in the study and responsibility for writing the manuscript. The corresponding author had final responsibility for the decision to submit for publication.

## Results

**Figure 1:**
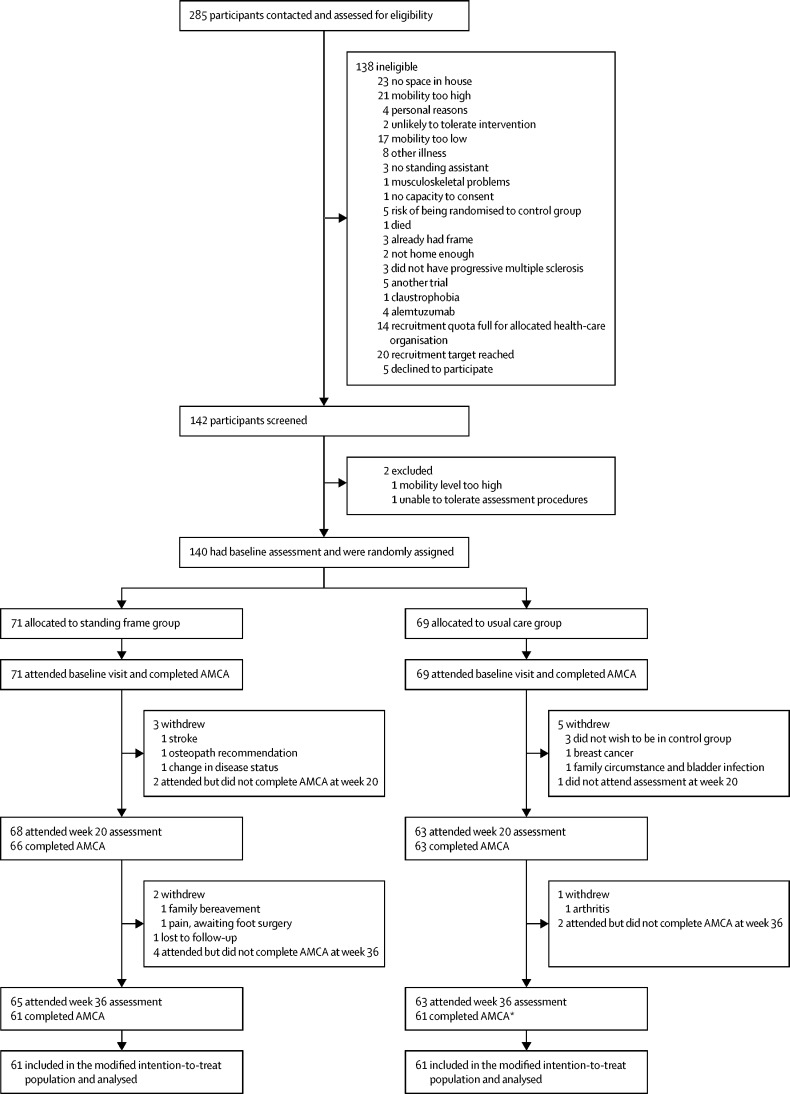
Trial profile AMCA=Amended Motor Club Assessment. *One participant did not attend the 20-week assessment but returned for week 36.

Between Sept 16, 2015, and April 28, 2017, we screened 285 potential participants. After screening, 140 participants were randomly assigned to either use a standing frame in addition to usual care (n=71) or to usual care alone (n=69; figure 1). Baseline characteristics were broadly consistent across the allocated groups (table 1). Some imbalances in sex and type of multiple sclerosis were observed: the proportion of men allocated to the standing frame group was higher than that allocated to the usual care group, and the proportion of participants with primary progressive multiple sclerosis was higher in the standing frame group than in the usual care group (table 1). Additionally, there was an imbalance in baseline AMCA score, with a lower mean score in the standing frame group compared to that in the usual care group (table 2).

**Table 1:** Demographic data and baseline characteristics

		**Standing frame group (n=71)**	**Usual care group (n=69)**	**All (n=140)**
Age, years		58·5 (51·3–66·4)	60·1 (54·1–66·0)	59·6 (52·6–66·2)
Mean EDSS score (SD; range)		7·3 (0·6; 6·5–8·0)	7·2 (0·6; 6·5–8·0)	7·3 (0·6; 6·5–8·0)
	6·5	24 (34%)	18 (26%)	42 (30%)
	7·0	11 (15%)	17 (25%)	28 (20%)
	7·5	11 (15%)	16 (23%)	27 (19%)
	8·0	25 (35%)	18 (26%)	43 (31%)
Sex				
	Men	31 (44%)	19 (28%)	50 (36%)
	Women	40 (56%)	50 (72%)	90 (64%)
Type of multiple sclerosis				
	Primary progressive	28 (39%)	16 (23%)	44 (31%)
	Secondary progressive	43 (61%)	53 (77%)	96 (69%)
Most recent relapse				
	>1 year	62 (87%)	63 (91%)	125 (89%)
	Within 3 months	2 (3%)	2 (3%)	4 (3%)
	Within 6 months	2 (3%)	0	2 (1%)
	Within 12 months	1 (1%)	2 (3%)	3 (2%)
	Unknown	4 (6%)	2 (3%)	6 (4%)
Occupation				
	Unemployed	5 (7%)	3 (4%)	8 (6%)
	Student	0	1 (1%)	1 (1%)
	Part-time work	2 (3%)	7 (10%)	9 (6%)
	Full-time work	1 (1%)	1 (1%)	2 (1%)
	Retired due to age	7 (10%)	8 (12%)	15 (11%)
	Medically retired	56 (79%)	49 (71%)	105 (75%)
Indoor walking aid				
	One stick	3 (4%)	2 (3%)	5 (4%)
	Two sticks	7 (10%)	8 (12%)	15 (11%)
	Frame	27 (38%)	30 (43%)	57 (41%)
	Wheelchair	47 (66%)	48 (70%)	95 (68%)
Outdoor walking aid				
	One stick	2 (3%)	2 (3%)	4 (3%)
	Two sticks	6 (8%)	6 (9%)	12 (9%)
	Frame	11 (15%)	15 (22%)	26 (19%)
	Wheelchair	67 (94%)	64 (93%)	131 (94%)
Wheelchair use				
	None	4 (6%)	4 (6%)	8 (6%)
	Occasionally	4 (6%)	3 (4%)	7 (5%)
	Monthly	2 (3%)	1 (1%)	3 (2%)
	Weekly	13 (18%)	10 (14%)	23 (16%)
	Daily	48 (68%)	51 (74%)	99 (71%)
Medical History				
	None of note	14 (20%)	13 (19%)	27 (19%)
	Osteoarthritis	6 (8%)	9 (13%)	15 (11%)
	Coronary heart disease or hypertension	15 (21%)	9 (13%)	24 (17%)
	Diabetes	8 (11%)	1 (1%)	9 (6%)
	COPD	6 (8%)	1 (1%)	7 (5%)
	Migraine	7 (10%)	5 (7%)	12 (9%)
	Other neurological condition	4 (6%)	3 (4%)	7 (5%)
	Depression	27 (38%)	30 (43%)	57 (41%)
	Osteoporosis	5 (7%)	8 (12%)	13 (9%)
	Other	25 (35%)	23 (33%)	48 (34%)

Data are n (%) or median (IQR), unless otherwise specified. EDSS=Expanded Disability Status Scale. COPD=chronic obstructive pulmonary disease.

**Table 2:** Primary outcome of Amended Motor Club Assessment (AMCA) scores at 36 weeks: primary modified intention-to-treat (mITT) analysis and Complier Average Causal Effect (CACE) sensitivity analyses

		**AMCA score for standing frame group (n=71)**		**AMCA score for usual care group (n=69)**		**AMCA score for compliers**		**AMCA score for non-compliers plus usual care group**		Fully adjusted analysis, mean difference (95% CI)*	**Analysis adjusted for baseline alone, mean difference (95% CI)**
		Baseline	Week 36	Baseline	Week 36	Baseline	Week 36	Baseline	Week 36		
mITT analysis		26·1 (13·9; 3·0–59·0; n=71)†	29·3 (17·2; 1·0–68·0; n=61)†	30·2 (14·6; 6·0–66·0; n=69)†	28·2 (17·0; 0·0–68·0; n=61)†	..	..	..	..	4·7 (1·9–7·5); p=0·0014	4·6 (1·6–7·6); p=0·0030
CACE analyses											
	Best 16 weeks	..	..	..	..	26·2 (13·7; 3·0–56·0; n=49)†	29·9 (16·0; 6·0–65·0; n=46)†	29·1 (14·6; 6·0–66·0; n=91)†	28·4 (17·5; 1·0–68·0; n=76)†	6·1 (2·5–9·8); p=0·00094	6·1 (2·2–9·9); p=0·0020
	Worst 16 weeks	..	..	..	..	28·2 (13·4; 8·0–56·0; n=36)†	31·6 (16·4; 8·0–65·0; n=35)†	28·1 (14·7; 3·0–66·0; n=104)†	27·9 (17·1; 1·0–68·0; n=87)†	7·9 (3·1–12·8); p=0·0013	7·9 (2·8–13·0); p=0·0025
	Weeks 5–20	..	..	..	..	26·7 (14·0; 3·0–56·0; n=46)†	30·5 (15·9; 6·0–65·0; n=43)†	28·8 (14·5; 6·0–66·0; n=94)†	28·1 (17·5; 1·0–68·0; n=79)†	6·5 (2·6–10·4); p=0·0010	6·5 (2·3–10·6); p=0·0022
	Best 32 weeks	..	..	..	..	26·6 (14·0; 3·0–56·0; n=46)†	32·4 (16·6; 6·0–65·0; n=43)†	32·4 (16·6; 6·0–65·0; n=43)†	28·0 (14·5; 6·0–66·0; n=94)†	6·5 (2·7–10·4); p=0·00077	6·5 (2·4–10·5); p=0·0016
	Worst 32 weeks	..	..	..	..	28·4 (13·9; 8·0–56·0; n=36)†	32·4 (16·6; 6·0–65·0; n=35)†	28·0 (14·5; 3·0–66·0; n=104)†	27·5 (16·9; 1·0–68·0; n=87)†	7·9 (3·1–12·7); p=0·0013	7·8 (2·8–12·9); p=0·0025
	Weeks 5–36	..	..	..	..	27·3 (13·8; 3·0–56·0; n=42)†	31·9 (15·7; 6·0–65·0; n=41)†	28·5 (14·6; 4·0–66·0; n=98)†	27·5 (17·4; 1·0–68·0; n=81)†	6·8 (2·8–10·8); p=0·00078	6·8 (2·6–11·0); p=0·0016

Data are mean (SD; range), unless otherwise specified. Mean differences in both analyses are between the standing and usual care group.

* Adjusted for baseline AMCA Score, region and Expanded Disability Status Scale category.

† n is the total number of participants who provided data at that timepoint.

At the primary endpoint, 36 weeks post-randomisation, the pooled (ie, across both groups) SD of the AMCA score was 16·9 points, with a correlation between baseline and week 36 AMCA score of 0·86. Individual-level changes in the score between baseline and week 36 assessments by allocated group are shown in appendix (p 10). The AMCA score at week 36 was significantly higher in the standing frame group than the usual care group, with a fully adjusted between-group mean difference of 4·7 points (95% CI 1·9–7·5, p=0·0014; table 2). Results of the analysis adjusted for baseline AMCA score alone were similar.

Analyses of 36-week AMCA subscores and short-term AMCA scores at 20 weeks showed significant fully adjusted between-group mean differences in favour of the standing frame group (appendix pp 11, 14). We observed short-term, statistically significant differences in favour of the standing frame group at 20 weeks in hip goniometry, knee extensor strength, and in both the physical and psychological components of the MSIS-29 scale (appendix pp 11–13). We also observed longer term significant differences, at 36 weeks, in hip and ankle goniometry in favour of the standing frame group; the short-term differences in MSIS-29 scale were not sustained at 36 weeks (appendix pp 14–16). The proportion of participants having two or more falls during weeks 21–36 was significantly lower in the standing frame group, with odds ratio of 0·43 (95% CI 0·20–0·94, p=0·035), but there was no significant between-group difference over weeks 1–20 or the full 36-week study period. Falling days per person-year, pooled across both groups, was 9·9 during 36 weeks.

18 serious adverse events were reported in 15 participants (seven participants in the usual care group and eight in the standing frame group; three participants each had two serious adverse events), none of which occurred during or in relation to the standing frame intervention. These serious adverse events were in line with expectations: urinary tract infections (n=8), cardiovascular events (stroke [n=2] and arrhythmia [n=1]), breast cancer (n=1), falls (n=3, of whom two participants fractured a hip), respiratory infections (n=2), and burns (n=1). In two individuals, pressure sores on the heels developed after hospital admission. For one of these participants, this resulted in the inability to continue using the frame after hospital discharge, despite regular use pre-hospitalisation.

Our adverse event reporting was based on so-called new symptoms, recorded with pre-formatted daily diaries, and is distinct from the serious adverse event data. Overall, 1924 symptoms were recorded (1188 in the standing frame group and 736 in the usual care group; table 3). These were expected in people with multiple sclerosis.^3^ We observed a disparity between the groups in the frequency of short-term musculoskeletal pains, such as aching leg muscles, which was potentially related to the intervention. The musculoskeletal pain lasted for longer than 7 days in five individuals (two in the standing frame group and three in the usual care group).

**Table 3:** Self-reported adverse events (new symptoms) according to allocated group

				**Standing frame group (n=71)**	**Usual care group (n=69)**
Adverse events lasting <7 days				1188	736
	Pain			551	180
		Categorised according to organ classification*			
			Musculoskeletal	486	160
			Neurological	16	12
			Abdominal	9	6
			Gynaecological	0	2
			Renal	2	0
			Respiratory	1	0
	Spasms			231	197
	Fatigue			60	184
	Urinary tract infection			45	36
	Numbness or sensory disturbance			41	33
	Tremor or shaking			7	24
	Weakness			24	23
	Constipation or diarrhoea			7	17
	Vertigo			22	9
	Virus			31	5
	Chest Infection			16	5
	Leg or back stiffness or tightness			23	2
	Headache			3	3
	Visual disturbance			3	3
	Seizures			0	2
	Balance problems			5	2
	Loss of bladder control			0	2
	Slurred speech			0	1
	Multiple sclerosis relapse			1	1
	Confusion			0	1
	Rash			0	1
	Toe infection			0	1
	Shingles			0	1
	Bladder spasms			2	1
	Blood in urine			0	1
	Nausea or vomiting			2	1
	Low sodium			1	0
	Ankle swelling			4	0
	Depression			1	0
	Shortness of breath			3	0
	Tennis elbow			1	0
	Low blood pressure			3	0
	Bruising			1	0
Participants reporting adverse events lasting ≥7 days (number of participants)				28	21
	Urinary tract infection			10	4
	Chest infection			10	5
	Nervous system			4	6
		Spasms		2	4
		Fatigue		2	1
		Weakness		0	1
		Stiff legs		1	0
	Bowel difficulties			0	3
	Infection			1	0
	Psychiatric (depression)			1	0
	Musculoskeletal pain†			2	3

* Pain categorised according to the MedDRA organ classification system.

† Usual care group: coccyx pain (lasting 18 days), heel pain (9 days), and hip pain (22 days); standing frame group: back pain (lasting 11 days) and joint ache (14 days).

**Figure 2:**
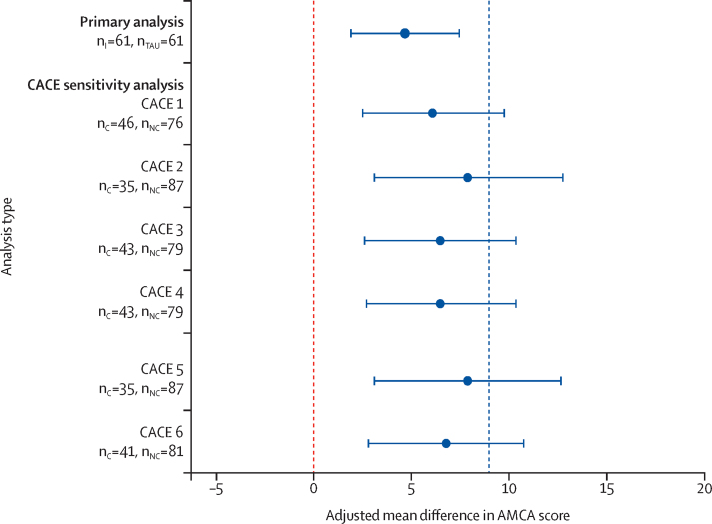
Fully adjusted mean difference in Amended Motor Club Assessment (AMCA) score at 36 weeks for the primary analysis and Complier Average Causal Effect (CACE) sensitivity analyses CACE sensitivity analyses done under the six compliance definitions (numbered 1–6). Error bars represent 95% CIs. The blue dashed line represents the pre-specified minimal clinically important difference of 9 points on the AMCA scale. n_I_=number of participants in the standing frame group. n_TAU_=number of participants in the usual care group. n_C_=number of compliers. n_NC_=number of compliers plus participants in the usual care group.

Prespecified sensitivity analyses of the primary outcome with additional adjustment for variables with observed baseline imbalance (sex and type of multiple sclerosis) were consistent with the primary analysis results. The planned CACE sensitivity analyses yielded results consistent with the primary analysis, although, under the CACE approach, the average between-group mean differences were larger and all the CIs included 9·0 (figure 2). The repeated-measures modelling gave similar results to the primary analysis, with a significant between-group difference in mean AMCA score at week 20 of 3·7 points (95% CI 1·2–6·2, p=0·004) and at week 36 of 4·5 points (2·0–7·0, p<0·001).

The estimated mean intervention cost per participant was £808 (SD 91; appendix p 17). The main cost drivers were the standing frame (£504) and physiotherapist home visits (£76). Mean costs to the NHS–PSS over the follow-up period (adjusted for cost at baseline, EDSS category, and region) were approximately £539 less for the standing frame group than for the usual care group, excluding the cost of the intervention itself. With the addition of the intervention cost, adjusted mean costs to the NHS–PSS were approximately £268 greater for the standing frame group (table 4, and appendix pp 18–26). The amount of informal care used by our study population was substantial, and application of a national average hourly rate to this time gave an adjusted informal care cost of approximately £3643 less in the standing frame group than in the usual care group (table 4; appendix pp 18–26). The mean EQ-5D-5L increase from baseline to 36-week follow-up was 0·042 for the standing frame group and 0·01 for the usual care group. This equated to an adjusted mean of 0·018 (95% CI −0·014 to 0·051) additional QALYs over the period of follow-up (table 4).

**Table 4:** Estimated costs and EQ-5D-5L values by group, and adjusted cost and adjusted quality-adjusted life-years (QALYs) differences, over a 36-week follow-up

		**Standing frame (n=71)**	**Usual care (n=69)**	Difference adjusted for baseline covariates*†
Resource item				
	Primary care	£594·58 (831·29); n=65	£470·46 (681·94); n=62	15·79 (−199·74 to 248·23)
	Secondary care	£1787·40 (4155·02); n=65	£2074·17 (3836·70); n=62	−284·82 (−1368·04 to 1077·62)
	Personal social services	£477·58 (1359·09); n=65	£947·28 (3086·93); n=62	−10·78 (−408·81 to 369·46)
	Total NHS–PSS (excluding standing frame intervention)	£2859·56 (4958·43); n=65	£3491·91 (5408·15); n=62	−539·27 (−1953·60 to 1138·40)
	Standing frame intervention	£807·74; n=54	..	..
	Total NHS–PSS	£3667·30 (4958·43); n=65	£3491·91 (5408·15); n=62	268·47 (−1093·79 to 2051·38)
	Patient personal costs	£2999·25 (6951·45); n=65	£2117·50 (3437·69); n=62	709·07 (−998·70 to 2469·58)
	Informal care	£16 047·16 (9944·57); n=65	£18 624·35 (13 589·22); n=62	−3643·34 (−6020·19 to −1348·18)
	Total costs (NHS, PSS, and patient and informal care)	£21 905·97 (12 147·65); n=65	£24 233·75 (13 464·93); n=62	−2192·41 (−5755·23 to −1163·43)
EQ-5D-5L values by timepoint				
	Baseline	0·224 (0·272, range −0·352 to 0·813); n=71	0·251 (0·274, range −0·265 to 0·778); n=69	..
	20 weeks	0·294 (0·269, range −0·256 to 0·813); n=68	0·271 (0·304, range −0·319 to 0·779); n=63	..
	36 weeks	0·266 (0·303, range −0·307 to 0·767); n=65	0·262 (0·293, range −0·358 to 0·836); n=62	..
QALYs (based on EQ-5D-5L) over the 36-week follow-up		0·189 (0·174, range −0·125 to 0·549); n=65	0·183 (0·182, range −0·142 to 0·544); n=62	0·018 (−0·014 to 0·051)

Data are mean (SD) or mean (95% CI), unless otherwise specified.

* Cost (specific to each cost component) or EQ-5D-5L value at baseline, Expanded Disability Status Scale category (≥7·5 to <7·5) at baseline, and region.

† Mean (95% CI) from bootstrap with 10 000 replication.

The cost-per-QALY of the intervention from the perspective of the NHS–PSS was approximately £14 700 (appendix pp 27). Uncertainty around this estimate is illustrated in the cost-effectiveness plane of bootstrapped replicates of incremental costs and incremental QALYs (appendix pp 28). These simulations suggested that, on 87% of occasions, the standing frame group would have greater QALYs over the period of follow-up than those of the usual care group. The bootstrap replicates also indicated a 0·52 probability of the intervention being considered cost-effective at a willingness-to-pay threshold of £20 000 per QALY and a 0·61 probability at a threshold of £30 000 per QALY. Broadening the analysis perspective beyond health and social care, in line with the recommendations of the Second Panel on Cost-Effectiveness in Health and Medicine,^26^ increased the apparent cost-effectiveness of the intervention.

There were few missing data and thus, we did not use multiple imputation. Sensitivity analyses explored the broader societal perspective and also took into account the 10-year life of the frames and the NHS's policy of equipment re-use. For both scenarios, the intervention appeared dominant in terms of cost-effectiveness (appendix pp 27–28).

## Discussion

Our results provide high-quality evidence that, compared with usual care alone, regular use of frame standing plus usual care provides significant improvements in motor function (our primary outcome) in people severely physically impaired with progressive multiple sclerosis, although not to the degree that was considered a priori as clinically meaningful. We also found evidence for differences in favour of the standing frame group regarding hip and ankle joint range and quality of life (secondary outcomes). This standing frame intervention was shown to be feasible for people with progressive multiple sclerosis to self-manage with the help of a standing assistant and for physiotherapists to implement within routine clinical practice.

Less clearcut is whether the outcome of the standing frame intervention was clinically meaningful. Interpretation is difficult because of the insufficient evidence to define what constitutes a minimal clinically important difference on the AMCA score. We relied on the only two physiotherapy studies we were aware of that had used the AMCA score; both suggested that a 9-point improvement was clinically relevant in people with severe multiple sclerosis.^14^, ^24^ A 9-point change could mean, for example, that a person could have improved so that they could balance in sitting to dress themselves (3 points), transfer independently (3 points), and stand without having to use their hands for balance (3 points). However, an improvement in any single one of these functional activities might constitute a clinically meaningful change. This view is supported by the audio narrative accounts of the changes undergone by SUMS study participants. When considering the design of future studies, further exploration is needed regarding the minimal clinically important difference on this measure for severely impaired individuals.

Our CACE analysis showed that accounting for compliance to the intervention resulted in a larger estimated intervention effect, with the prespecified minimal clinically important difference of 9 points on the AMCA score contained within the 95% CIs of all six compliance definitions. This suggests a positive association between compliance with the intervention and the motor benefits gained. This is consistent with theoretical expectations and with the results of (low methodological quality) studies of standing frame use in populations with other neurological conditions.^6^

To sustain any benefits gained from physical activity, individuals need to maintain long-term engagement, which is a particular challenge for people with a disability.^27^ Evidence is scarce regarding long-term adherence in people with multiple sclerosis to physical activity interventions; however, non-adherence rates are as high as 80% for individuals with chronic conditions for which interventions might aim to slow down decline rather than to cure.^27^ Two thirds of the participants in the standing frame group continued to stand regularly in the frame during the 36-week period, which, in light of the literature, we consider to be a high proportion. Furthermore, 70% of participants who had a standing frame during the study requested to keep the frame on completing the study, thus further supporting the feasibility and acceptability of the intervention.

Behavioural change techniques were an integral component of the standing frame intervention. To complement the physiotherapy advice and support, individuals had access to paper-based, DVD, and online resources, designed to equip them and their standing assistants with the knowledge and skills necessary to undertake this activity within their own homes. Aimed at enhancing self-efficacy,^22^ this approach was considered essential because self-efficacy is a key determinant of physical activity behaviour in people with multiple sclerosis^28^ and is typically low.^12^

Tolerability of an intervention is important for adherence and thus, capturing adverse events potentially associated with the intervention was important. We achieved this by using daily, self-reported, preformatted diaries. However, free-text description of adverse events was often ambiguous, making it difficult to determine whether they were new symptoms. Therefore, it is challenging to precisely state what proportion of these broad-ranging symptoms are related to the standing frame intervention. Bias in reporting of adverse events is also possible because the standing frame group recorded both details of each standing session and any new symptoms in the same diaries, potentially triggering reporting of new symptoms more comprehensively than in the usual care group. However, overall, the data suggest that this intervention is well tolerated; the adverse events were typically transient (lasting less than 7 days), musculoskeletal in nature (aches and pains), and occurred early in the programme when participants were probably adjusting to recommencement of regular standing. Importantly, physiotherapists should inform people that short-term musculoskeletal aches and pains might occur and provide education about how to manage this. From a methodological perspective, effective and reliable systems for collecting adverse event data in rehabilitation trials should be further investigated.

Our study has several strengths. To our knowledge, this is the largest randomised controlled physical rehabilitation study to date undertaken in severely impaired people with progressive multiple sclerosis. It was the first definitive multicentre randomised controlled trial to assess the clinical and cost effectiveness, safety, and tolerability of a home-based, self-managed standing frame programme in this population. The study was originally planned to have 80% power, on the basis of conservative assumptions;^16^ with our observed SD being lower and the correlation between baseline and week 36 AMCA scores higher than anticipated, we were able to estimate the intervention effect with increased precision. Our cost-effectiveness analysis assumed that a new standing frame would be purchased for everyone in the intervention group; however, given the NHS policy of equipment re-use, and the average 10-year life of a frame, our cost-effectiveness estimate is likely to be conservative.

Another strength of our study is that it was a pragmatic trial. To maximise generalisability of the results, we minimised our exclusion criteria. The intervention was delivered by physiotherapists working within the NHS, who did not undergo specific training for this intervention, making it likely that similar results would be gained on implementation within usual practice. However, we should note that our findings cannot automatically be generalised to other countries that do not have a similar organisational context. The publication of our educational resources on a freely available website aims to enhance shared, evidence-based, decision making about the effect of introducing this intervention to people's daily lives.

Our study has several limitations. Our primary economic outcome measure was QALYs, in line with guidance by the National Institute for Health and Care Excellence. The difference in EQ-5D-5L scores (used to calculate QALYs) between the standing frame and usual care groups at 36-weeks did not reach the minimal clinically important difference for the EQ-5D-5L score described by Marra and colleagues.^29^ Therefore, it could be argued that the QALY gain was not perceptibly different from zero, implying that the intervention was not cost-effective. However, the standing frame intervention did appear effective from the patient's perspective when considered across outcome measures, specifically according to the primary clinical outcome measure. Our main analysis might have been restrictive in identifying benefits of the intervention, and a broader societal perspective might have been preferable.

The usual care group was not offered an intervention and hence, we could not exclude that placebo effects might have contributed to the benefits experienced by the standing frame group. However, the primary outcome was clinician-rated and measured by a masked assessor, which should reduce the effect of this. Nevertheless, further research is needed to disentangle the intrinsic effects of standing from non-specific effects due to, for example, attention. It is also possible that drug interventions might have contributed to any of the changes observed. However, participants were excluded if there had been any recent changes in disease-modifying therapies, and they were asked to record any medication changes throughout the study period; the two groups were balanced in terms of medication changes, therefore, this is unlikely to account for the between-group differences.

In conclusion, there is a paucity of evidence-based, self-management interventions that are recommended for people severely impaired with progressive multiple sclerosis who have few treatment options available. We hope this intervention can now be offered and reimbursed more widely as a management option for this population.

## Data sharing

The SUMS study protocol and statistical analysis plan are publicly available at https://www.plymouth.ac.uk/research/sums. Individual participant data that underlie the results will be made available (after de-identification) on a controlled access basis, subject to suitable data sharing agreements. Requests for data sharing should be made to the Chief Investigator (CI; J Freeman) in the first instance. Requesters will be asked to complete an application form detailing specific requirements, rationale, and proposed usage. Requests will be reviewed by the CI and study sponsor, who will consider the viability and suitability of the request and the credentials of the requester. Where access to requested data is granted, requesters will be asked to sign a data sharing agreement. Requested data will be made available, along with supporting documentation (eg, data dictionary) on a secure server or through other secure data transfer method.
