# Supplementary material for: Assessment of a home-based standing frame programme in people with progressive multiple sclerosis (SUMS): a pragmatic, multi-centre, randomised, controlled trial and cost-effectiveness analysis
Source: Lancet Neurol. 2019 Aug;18(8):736–47. doi: 10.1016/S1474-4422(19)30190-5 (PMC7646281; doi:10.1016/S1474-4422(19)30190-5)

THE LANCET
Neurology

Supplementary appendix

This appendix formed part of the original submission and has been peer reviewed. We post it as supplied by the authors.

Supplement to: Freeman J, Hendrie W, Jarrett L, et al. Assessment of a home-based standing frame programme in people with progressive multiple sclerosis (SUMS): a pragmatic, multi-centre, randomised, controlled trial and cost-effectiveness analysis. *Lancet Neurol* 2019; **18:** 736–47.

Evaluation of a home-based standing frame programme in people with progressive Multiple Sclerosis (SUMS): a pragmatic, multi-centre, randomised, controlled trial and cost-effectiveness analysis.

Contents of the Appendix:

Figure/Table Title	Page number
Table A1: Pre-specified definitions which could trigger Complier Average Causal Effect (CACE) analyses	2
Cost–effectiveness analysis – detailed methods	3-4
Table A2: UK nationally recognised unit costs in British pounds sterling	5-9
Figure A1: Histograms of individual-level change in AMCA score between baseline and week 36 assessments by allocated group.	10
Table A3: Secondary Outcomes at Week 20	11-13
Table A4: Secondary Outcomes at Week 36	14-16
Table A5: - Standing frame intervention costs (per participant)	17
Table A6: Resource use and cost data over 36 week follow-up and cost data at baseline by allocated group	18-26
Table A7: Incremental cost-effectiveness analyses and sensitivity analyses	27
Figure A2: The cost-effectiveness plane of bootstrapped replicates of incremental costs and incremental QALYs	28

Table A1: Pre-specified definitions which could trigger Complier Average Causal Effect (CACE) analyses

Definition 1	Total of at least 1152 minutes standing, assessed over the participant’s best 16 weeks during weeks 1-20
Definition 2	Total of at least 1152 minutes standing, assessed over the participant’s worst 16 weeks during weeks 1-20
Definition 3	Total of at least 1152 minutes standing, assessed over weeks 5-20
Definition 4	Total of at least 2304 minutes standing, assessed over the participant’s best 32 weeks
Definition 5	Total of at least 2304 minutes standing, assessed over the participant’s worst 32 weeks
Definition 6	Total of at least 2304 minutes standing, assessed over weeks 5-36

“Best” and “worst” weeks were determined according to most and least total stand time, respectively. The rationale for the cut-off’s for these definitions is detailed in the Statistical Analysis Plan, available at https://www.plymouth.ac.uk/research/sums

Cost–effectiveness analysis – detailed methods

A within-trial cost-effectiveness analysis was conducted. This estimated the additional costs of delivering the intervention, costs associated with health, social care, carer and patient resource use, and quality-adjusted life-years (QALYs) over the 36 week trial period. The primary perspective was the UK NHS and Personal Social Services (PSS), with a broader societal perspective considered in sensitivity analyses.

Participant level data were recorded for length of physiotherapists' visits, telephone calls and travel time, in addition to distance travelled and time in non-contact activities. Other resources required for the delivery of the intervention were identified via questionnaire and discussion with the intervention developers. Self-report health, social and informal care resource use and participant expenses were collected via interviewer-administered questionnaires at baseline, 20-week and 36-week follow-up. Resource use data were combined with nationally recognised unit costs[1,2] in British pounds sterling (£, 2016) (see table A2 below). The primary economic endpoint was QALYs at 36-weeks. QALYs were estimated using self-report EQ-5D-5L data collected at baseline, 20-week and 36-week follow-up, and applying the 'cross-walk' algorithm[3] to provide QALY weights from the UK general population valuation survey of the EQ-5D-3 L[4]. This approach adheres to the current 'position statement' of the National Institute of Health and Care Excellence (NICE) regarding use of the EQ-5D.

Quantities of each component of intervention resource use were calculated, unit costs applied, and the mean (SD) intervention cost per participant estimated. Resource use quantities, by item, at baseline and 36-weeks were calculated. Unit costs were applied, and mean (SD) costs for each item calculated, by allocated treatment group. QALY estimates, for each group, were derived by application of the area-under-the-curve method, and linearly extrapolating from the baseline to 20-week assessment, and the 20-week to 36-week assessment, assuming a change in QALY weight halfway through each of these time periods.[5] The length of time was multiplied by the relevant QALY weight for each time period to obtain the number of QALYs for each group. No discounting was applied for costs or benefits, given the 36-week time horizon.

Generalised linear regression models (GLM) (with appropriate family and link functions) were used to estimate mean differences between the two groups in terms of costs and QALYs. Analyses were also conducted with adjustment for costs/EQ-5D-5L values at baseline and the pre-specified covariates, EDSS category (≤7.0 or ≥7.5) and region. Non-parametric bootstrapping with 10,000 replications, was used to estimate 95% CIs around the cost and QALY differences[5]. Incremental cost-effectiveness ratios (ICERs) were calculated ($Cost_{intervention}$ –$Cost_{control}$] / [$QALY_{intervention}$ – $QALY_{control}$), and the NICE willingness to pay threshold per additional QALY gained of £20,000 to £30,000[6] applied. The cost-effectiveness plane was used to present combinations of incremental costs and incremental QALYs from bootstrap replicates. Data were analysed on an ITT basis and the extent of missing data was explored with the intention of using regression-based multiple imputation as required in sensitivity analyses. A number of uncertainties were addressed in sensitivity analyses.

References

1. Curtis L, Burns A. (2016) *Unit Costs of Health & Social Care*. Personal Social Services Research Unit, Canterbury.

2. NHS Improvement. *NHS Reference Costs* 2016-17. https://improvement.nhs.uk/resources/reference-costs/, released 24/11/2017, accessed 24/08/2018.

3. van Hout B, Janssen MF, et al. Interim scoring for the EQ-5D-5L: Mapping the EQ-5D-5L to EQ-5D-3L value sets. *Value in Health* 2012;**15** (5):708-15.

4. Dolan P. Modelling valuations for EuroQol health states*. Med Care*. 1997; **35**(11): 1095-108.

5. Drummond M, Schulpher M, Torrance G, O'Brien B, Stoddart G. *Methods for the Economic Evaluation of Health Care Programmes.* 3rd Edition. Oxford University Press, 2005.

6. National Institute for Health and Care Excellence (NICE). *Guide to the Methods of Technology Appraisal* 2013. https://www.nice.org.uk/process/pmg9/resources/guide-to-the-methods-of-technology-appraisal-2013-pdf-2007975843781, published 04/04/2013, accessed 28/08/2018.

Table A2: UK nationally recognised unit costs in British pounds sterling

Resource item	***Unit cost*** ***(£, 2016)***	***Source of cost estimate***	***Basis of cost estimate***
**Intervention resources**			
Oswestry standing frame	£504.00	Theo Davies and Sons Ltd (frame manufacturers) (http://www.oswestry-frames.co.uk)	10% discounted from list price (£560) for study. Expectation that such discount also available to NHS.
Delivery cost for frame	£55.00	Theo Davies and Sons Ltd (frame manufacturers) (http://www.oswestry-frames.co.uk)	
Physiotherapist time to order frame (approx. 10-15 minutes)	£8.75	PSSRU, 2016, p.137	Band 6 Scientific and Professional staff, annual salary £31,351. Cost per working hour.
Intervention booklet and DVD	£2.00	Intervention developer	
Physiotherapist time to provide intervention	£42 per hour	PSSRU, 2016, p.137	Band 6 Scientific and Professional staff, annual salary £31,351. Cost per working hour.
Physiotherapist travel costs	Included in physiotherapist costs	PSSRU, 2016, p.136	Included in overhead costs for physiotherapist.
**Primary care**			
GP contacts (surgery)	£27.00	PSSRU 2016, p.145	Surgery consultation, 9.22 minutes.

GP contacts (home)	£34.20	PSSRU 2016, p.145<br><br>PSSRU 2015, p.176	Per minute of patient contact = £3 (allows for average of 12 minutes travel time per visit).<br><br>Home visit, 11.4 minutes.
GP telephone calls	£21.30	PSSRU 2016, p.145<br><br>PSSRU 2015, p.176	Per minute of patient contact = £3.<br><br>Telephone call, 7.1 minutes.
MS specialist nurse contacts (home)	£68.00	NHS Reference costs 2016/2017	Community Health Services, Other Specialist Nursing, Adult, face to face.
MS specialist nurse telephone calls	£33.00	NHS Reference costs 2016/2017	Community Health Services, Other Specialist Nursing, Adult, non-face to face.
Physiotherapist contacts (home)	£53.00	NHS Reference costs 2016/2017	Community Health Services, Physiotherapist, Adult, one to one.
Occupational therapist contacts (home)	£77.00	NHS Reference costs 2016/2017	Community Health Services, Occupational Therapist, Adult, one to one.
Practice nurse contacts (surgery)	£9.30	PSSRU 2016, p.143<br><br>PSSRU 2015, p.174	£36 per hour.<br><br>15.5 minute consultation.
Community nurse contacts (home)	£37.00	NHS Reference costs 2016/2017	Community Health Services, District Nurse, Adult face to face.
Chiropodist/Podiatrist contacts (surgery)	£41.00	NHS Reference costs 2016/2017	Community Health Services, Podiatrist, Tier 1, General Podiatry.
Continence advisor contacts	£83.00	NHS Reference costs 2016/2017	Community Health Services, Specialist Nursing, Adult face to face.
Community psychiatric nurse contacts (home)	£36.00	PSSRU 2016, p.142	£36 per hour.<br><br>1 hour visit

Counsellor contacts	£44.00	PSSRU 2016, p.137	Band 6 Scientific and Professional staff, £44 per hour. 1 hour consultation.
**Secondary care**			
Hospital stays (nights)	£455.17	NHS Reference costs 2016/2017	Medical care of patients with MS, non-elective stays (mean across Complication and Comorbidities scores by mean length of stay, £3,420/7.51).
Visits to A&E	£147.80	NHS Reference costs 2016/2017	Outpatient attendances data, Accident and Emergency.
Days in hospital	£369.00	NHS Reference costs 2016/2017	Medical care of patients with MS, day cases (mean across Complication and Comorbidities scores).
Rehabilitation unit stays (days)	£98.73	PSSRU 2016, p.197<br>PSSRU 2014, p.40	Community rehabilitation unit, £691.13 per person, per week. 2014 cost of £671 uprated by 3% based on PSS annual percentage increases for adult service (all sectors - pay & prices including capital).
Neurologist	£167.50	NHS Reference costs 2016/2017	Outpatient attendances data, Neurology.
MS specialist nurse	£44.00	PSSRU 2016, p.188	Hospital-based nurses. Band 6 Scientific and Professional staff, £44 per hour.
Occupational therapist	£64.99	NHS Reference costs 2016/2017	Outpatient attendances data, Occupational Therapy.
Physiotherapist	£48.81	NHS Reference costs 2016/2017	Outpatient attendances data, Physiotherapy.
Opthalmologist	£55.99	NHS Reference costs 2016/2017	Outpatient attendances data, Medical Opthalmology.
Orthotist	£119.07	NHS Reference costs 2016/2017	Outpatient attendances data, Orthotics.

Chiropodist	£46.64	NHS Reference costs 2016/2017	Outpatient attendances data, Podiatry.
Speech therapist	£96.52	NHS Reference costs 2016/2017	Outpatient attendances data, Speech and language therapy.
Psychologist	£168.65	NHS Reference costs 2016/2017	Outpatient attendances data, Clinical Psychology.
Psychiatrist	£142.00	NHS Reference costs 2016/2017	Mental Health,<br><br>Other psychiatric liaison services, Adult and Elderly.
Pain clinic	£139.23	NHS Reference costs 2016/2017	Outpatient attendances data, Pain management.
Urologist	£109.40	NHS Reference costs 2016/2017	Outpatient attendances data, Urology.
New wheelchair	£191	PSSRU 2016, p.96	Per active user chair per year.
Medication use (weeks of use):			
Disease-modifying medicine	£163.50	British National Formulary 70, p.730	Multiple Sclerosis, Interferon beta. Once weekly injection. 12 pre-filled injections = £1,962.
Botulinum toxin injections	£46.07	British National Formulary 70 p.324	Total dose of 400 units in 3 month period. Botox Allergan 200 unit powder for solution for injection vials. £276.40.
Phenol injections	£4.90	British National Formulary 70, p.81<br><br>Jarrett paper	5% phenol in glycerol, between 1.5 and 2.5 ml. 10 x 5ml ampoules = £47.91-£50.00. Doses at least six weeks apart (mean of 3 doses).
Intravenous steroids	£58.00	British National Formulary 70, p.584	Intravenous methylprednisolone:<br><br>500mg to 1g daily, for 3 to 5 days.<br><br>500mg vial methylprednisolone powder with solvent, £9.60; 1g vial, £17.30.<br><br>Requires 3 x 500mg vials to 5 x 1g vials, £28.80 to £86.50.

Steroid tablets	£145.00	British National Formulary 70, p.584	Oral methylprednisolone:<br>500mg to 2g daily, for 3 to 5 days.<br>20 x 100mg tablet pack, £48.32.<br>15 to 100 tablets requires 1 to 5 packs, £48.32 to £241.60.
**Social care**			
Home care worker contacts	£6.90	PSSRU 2016, p.160	Mean hourly cost of all home care. £18 per hour. 23 minute visit.
Social worker/Care manager contacts	£55.00	PSSRU 2016, p.156	Per hour of client-related work.
Respite unit/facility stays (day)	£141.00	PSSRU 2016, p.65	Local authority own-provision care homes for adults requiring physical support.
Day care centre (days)	£87.00	PSSRU 2016, p.67	Day care for adults requiring physical support.
**Informal care**	£13.79	Office for National Statistics, 'EARN08: Distribution of gross hourly earnings of employees: People, April to June 2016'.<br>Published 16/11/16.	https://www.ons.gov.uk/employmentandlabourmarket/peopleinwork/earningsandworkinghours/datasets/distributionofgrosshourlyearningsofemployeesearn08/current. Accessed 28/3/18.<br>Hourly estimate of cost of friend's/relative's time.
**Own expenses**			
Participant report		Participant self-report Resource Use Questionnaire'	Participant own estimate'

*Personal Social Services Research Unit, 'Unit Costs of Health and Social Care'.

Figure A1: Histograms of individual-level change in AMCA score between baseline and week 36 assessments by allocated group.

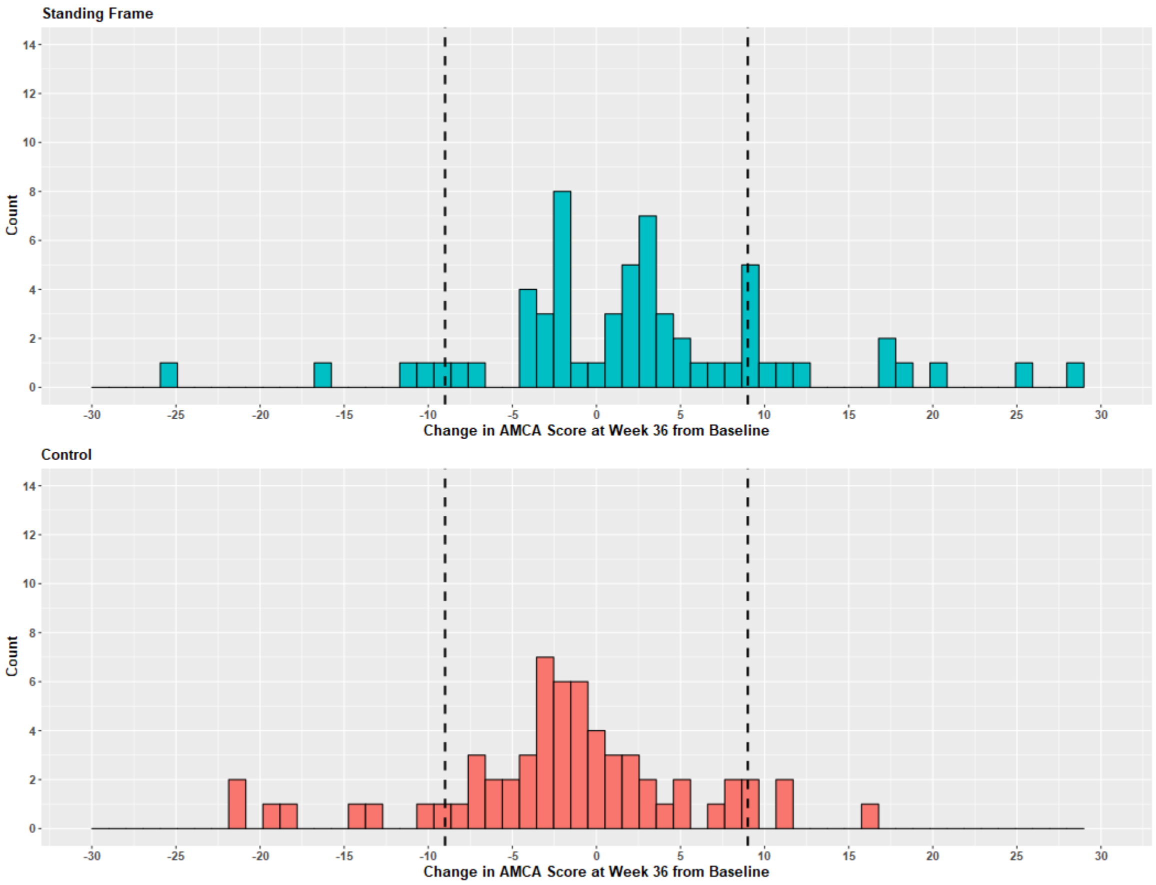

Vertical dashed lines represent worsening or improvement in AMCA of 9 points

Table A3: Secondary Outcomes at Week 20

	Mean (sd) [range]				Fully Adjusted Analysis[1]	Analysis Adjusted for Baseline only
	Standing Frame (n=71)		Usual Care (n=69)		Mean Difference (Standing – Usual Care) (95% CI)	Mean Difference (Standing – Usual Care) (95% CI)
	Baseline	Week 20	Baseline	Week 20		
	(n=69)[2]	(n=66)[2]	(n=69)[2]	(n=63)[2]		
AMCA	26.1 (13.9) [3.0, 59.0]	28.7 (16.3) [5.0, 67.0]	30.2 (14.6) [6.0, 66.0]	30.3 (16.7) [4.0, 63.0]	3.6 (1.3, 5.9) p=0.002	3.5 (1.1, 5.8) p=0.004
	(n=69)[2]	(n=64)[2]	(n=69)[2]	(n=63)[2]		
Hip Goniometry	5.5 (9.8) [-15.0, 25.0]	11.7 (6.7) [-7.5, 23.5]	4.1 (9.2) [-20.0, 25.0]	7.3 (8.0) [-21.5, 21.0]	3.7 (1.7, 5.8) p=0.001	3.6 (1.5, 5.7) p=0.001
	(n=71)[2]	(n=66)[2]	(n=68)[2]	(n=62)[2]		
Ankle Goniometry	-6.0 (10.5) [-37.5, 22.5]	0.2 (8.9) [-19.0, 21.0]	-3.1 (9.3) [-30.0, 15.0]	1.1 (7.8) [-16.5, 20.0]	1.1 (-1.2, 3.4) p=0.338	1.0 (-1.4, 3.4) p=0.400

	**(n=71)[2]**	**(n=66)[2]**	**(n=69)[2]**	**(n=63)[2]**		
Popliteal Goniometry	137.8 (14.7) [97.5, 180.0]	140.1 (15.2) [110.0, 195.0]	135.7 (26.5) [-30.0, 176.0]	137.9 (18.9) [46.5, 175.0]	2.7 (-1.9, 7.3) p=0.248	2.3 (-2.4, 7.0) p=0.329
	**(n=71)[2]**	**(n=65)[2]**	**(n=69)[2]**	**(n=62)[2]**		
Knee Extensors	123.5 (59.2) [16.5, 274.7]	152.0 (86.3) [17.5, 429.5]	141.2 (73.0) [12.8, 377.5]	143.5 (87.6) [0.0, 349.8]	25.1 (4.0, 46.2) p=0.020	22.4 (-1.3, 46.2) p=0.064
	**(n=70)[2]**	**(n=65)[2]**	**(n=69)[2]**	**(n=63)[2]**		
Functional Reach in Sitting	31.8 (13.9) [0.0, 59.5]	33.3 (13.7) [0.0, 58.0]	31.9 (13.2) [1.5, 59.5]	32.2 (13.1) [0.0, 65.0]	1.4 (-1.6, 4.4) p=0.359	1.3 (-1.7, 4.4) p=0.392
	**(n=71)[2]**	**(n=66)[2]**	**(n=69)[2]**	**(n=63)[2]**		
Forced Expiratory Volume	2.2 (0.7) [0.6, 3.9]	2.3 (0.7) [0.6, 4.1]	2.1 (0.7) [1.1, 4.7]	2.1 (0.8) [0.9, 4.9]	0.1 (-0.1, 0.2) p=0.385	0.1 (-0.1, 0.2) p=0.389
	**(n=71)[2]**	**(n=68)[2]**	**(n=69)[2]**	**(n=63)[2]**		
MSIS-29 - Physical	66.5 (19.6)	56.0 (22.0)	66.9 (20.2)	62.6 (21.4)	-6.8	-6.8

	[13.3, 100.0]	[3.3, 96.7]	[3.3, 96.7]	[5.0, 91.7]	(-12.5, 1.2)<br>p=0.019	(-12.4, -1.1)<br>p=0.020
	**(n=71)[2]**	**(n=68)[2]**	**(n=69)[2]**	**(n=63)[2]**		
MSIS-29 - Psychological	66.5 (19.6)<br>[13.3, 100]	34.5 (22.4)<br>[0.0, 96.3]	35.9 (21.1)<br>[0.0, 78.8]	39.2 (23.0)<br>[3.7, 85.2]	-7.7<br>(-13.4, -1.9)<br>p=0.009	-7.8<br>(-13.6, -2.0)<br>p=0.009
	**(n=71)[2]**	**(n=68)[2]**	**(n=69)[2]**	**(n=63)[2]**		
Bladder Control Scale	8.0 (6.5)<br>[0.0, 21.0]	7.3 (5.9)<br>[0.0, 20.0]	9.5 (6.6)<br>[0.0, 20.0]	8.1 (6.0)<br>[0.0, 22.0]	0.0<br>(-1.7, 1.8)<br>p=0.961	0.0<br>(-1.8, 1.8)<br>p=0.995
	**(n=71)[2]**	**(n=67)[2]**	**(n=68)[2]**	**(n=62)[2]**		
Bowel Control Scale	5.3 (5.7)<br>[0.0, 19.0]	4.7 (4.9)<br>[0.0, 19.0]	5.6 (5.3)<br>[0.0, 23.0]	5.3 (5.3)<br>[0.0, 23.0]	-0.2<br>(-1.5, 1.0)<br>p=0.716	-0.2<br>(-1.5, 1.1)<br>0.772

[1] Adjusted for baseline score, region and EDSS category; [2] n is the total number of participants who provided data at that time point

Table A4: Secondary Outcomes at Week 36

	Mean (sd) [range]				Fully Adjusted Analysis[1]	Analysis Adjusted for Baseline only
	Standing Frame (n=71)		Usual Care (n=69)		Mean Difference (Standing – Usual Care) (95% CI)	Mean Difference (Standing – Usual Care) (95% CI)
	Baseline	Week 36	Baseline	Week 36		
	(n=69)[2]	(n=55)[2]	(n=69)[2]	(n=61)[2]		
	(n=71)[2]	(n=61)[2]	(n=69)[2]	(n=61)[2]		
AMCA sub-score – Lower Limb	8.5 (6.8) [0, 25]	9.1 (7.4) [0, 25]	10.9 (7.2) [0, 28]	9.9 (7.9) [0, 28]	1.5 (0.4, 2.7) p=0.009	1.6 (0.5, 2.7) p=0.006
	(n=71)[2]	(n=61)[2]	(n=69)[2]	(n=61)[2]		
AMCA sub-score – Functional Movement	17.6 (8.0) [3, 36]	20.1 (10.6) [1,45]	19.3 (8.4) [4, 40]	18.8 (9.9) [4,42]	3.0 (0.9, 5.1) p=0.005	2.8 (0.5, 5.1) p=0.017
Hip Goniometry	5.5 (9.8) [-15.0, 25.0]	11.6 (7.9) [-17.5, 22.5]	4.1 (9.2) [-20.0, 25.0]	7.7 (6.5) [-10.0, 20.0]	2.8 (0.6, 4.9) p=0.012	2.7 (0.6, 4.9) p=0.013

	**(n=71)²**	**(n=59)²**	**(n=68)²**	**(n=60)²**		
Ankle Goniometry	-6.0 (10.5) [-37.5, 22.5]	2.4 (10.0) [-22.5, 24.0]	-3.1 (9.3) [-30.0, 15.0]	1.8 (9.1) [15.0, 25.0]	3.1 (0.6, 5.7) p=0.015	2.7 (-0.1, 5.6) p=0.058
	**(n=71)²**	**(n=59)²**	**(n=69)²**	**(n=61)²**		
Popliteal Goniometry	137.8 (14.7) [97.5, 180.0]	139.5 (16.4) [92.5, 197.5]	135.7 (26.5) [-30.0, 176.0]	140.6 (15.9) [95.0, 177.5]	-0.5 (-4.8, 3.7) p=0.799	-0.9 (-5.2, 3.4) p=0.675
	**(n=71)²**	**(n=60)²**	**(n=69)²**	**(n=61)²**		
Knee Extensors	123.5 (59.2) [16.5, 274.7]	154.9 (99.4) [0.0, 420.0]	141.2 (73.0) [12.8, 377.5]	151.1 (90.4) [0.0, 361.7]	18.4 (-5.5, 42.3) p=0.130	14.8 (-12.0, 41.7) p=0.277
	**(n=70)²**	**(n=62)²**	**(n=69)²**	**(n=61)²**		
Functional Reach in Sitting	31.8 (13.9) [0.0, 59.5]	32.5 (14.8) [0.0, 60.5]	31.9 (13.2) [1.5, 59.5]	29.8 (12.7) [0.0, 54.5]	2.6 (-1.1, 6.3) p=0.166	2.5 (-1.3, 6.2) p=0.196
	**(n=71)²**	**(n=64)²**	**(n=69)²**	**(n=63)²**		
Forced Expiratory Volume	2.2 (0.7)	2.2 (0.7)	2.1 (0.7)	2.2 (0.8)	-0.1	-0.1

	[0.6, 3.9]	[0.9, 3.9]	[1.1, 4.7]	[0.9, 4.8]	(-0.2, 0.1)<br>p=0.425	(-0.2, 0.1)<br>p=0.364
	**(n=71)[2]**	**(n=65)[2]**	**(n=69)[2]**	**(n=63)[2]**		
MSIS-29 - Physical	66.5 (19.6)<br>[13.3, 100.0]	57.9 (23.6)<br>[0.0, 98.3]	66.9 (20.2)<br>[3.3, 96.7]	62.1 (21.4)<br>[10.0, 93.3]	-3.7<br>(-10.0, 2.5)<br>p=0.239	-3.7<br>(-9.9, 2.5)<br>p=0.245
	**(n=71)[2]**	**(n=65)[2]**	**(n=69)[2]**	**(n=63)[2]**		
MSIS-29-Psychological	66.5 (19.6)<br>[13.3, 100]	37.9 (22.9)<br>[0, 85.2]	35.9 (21.1)<br>[0.0, 78.8]	36.2 (24.4)<br>[0, 92.6]	-0.7<br>(-6.8, 5.4)<br>p=0.827	-0.7<br>(-6.7, 5.4)<br>p=0.828
	**(n=71)[2]**	**(n=65)[2]**	**(n=69)[2]**	**(n=63)[2]**		
Bladder Control Scale	8.0 (6.5)<br>[0.0, 21.0]	7.5 (6.4)<br>[0.0, 22.0]	9.5 (6.6)<br>[0.0, 20.0]	8.3 (6.4)<br>[0.0, 22.0]	0.3<br>(-1.4, 2.1)<br>p=0.720	0.3<br>(-1.5, 2.0)<br>p=0.765
	**(n=71)[2]**	**(n=65)[2]**	**(n=68)[2]**	**(n=62)[2]**		
Bowel Control Scale	5.3 (5.7)<br>[0.0, 19.0]	5.4 (5.7)<br>[0.0, 21.0]	5.6 (5.3)<br>[0.0, 23.0]	5.7 (5.3)<br>[0.0, 19.0]	0.01<br>(-1.6, 1.6)<br>p=0.989	0.03<br>(-1.6, 1.6)<br>p=0.971

[1] Adjusted for baseline score, region and EDSS category; [2] n is the total number of participants who provided data at that time point

Table A5: - Standing frame intervention costs (per participant)

Resource item	***N***	***Mean cost (£)***	***SD cost (£)***	***Min cost (£)***	***Max cost (£)***
Standing frame	71	504.00	-	-	-
Delivery charge for frame	71	55.00	-	-	-
Physiotherapist time to order frame	71	8.75	-	-	-
Intervention booklet and DVD	71	2.00	-	-	-
Physiotherapist visits (contact time)	66	76.30	25.40	38.50	147.00
Physiotherapist visits (non-contact time)	67	27.0	19.90	3.50	84.00
Physiotherapist telephone calls (contact time)	59	46.76	16.55	18.90	93.10
Physiotherapist telephone calls (non-contact time)	56	32.53	20.06	0	87.50
Telephone calls to physiotherapists	71	1.22	4.08	0	24.50
Additional non-contact time	71	5.84	15.24	0	84.00
Physiotherapist travel time	67	54.40	33.81	7.00	192.50
**Total cost**	**54**	**807.74**	**91.27**	**664.25**	**1075.85**

Table A6: Resource use and cost data over 36 week follow-up and cost data at baseline by allocated group

	Standing frame intervention		*Usual care*	
Resource use	**n**	**Mean (SD) [range]**	**n**	**Mean (SD) [range]**
**Primary care**				
GP contacts (surgery)	65	2.46 (3.75) [0-20]	62	2.11 (3.00) [0-19]
GP contacts (home)	65	0.52 (1.38) [0-9]	62	0.40 (1.25) [0-8]
GP telephone calls	65	2.03 (2.51) [0-10]	62	1.68 (2.21) [0-12]
MS specialist nurse contacts (home)	65	0.45 (0.83) [0-3]	62	0.27 (0.77) [0-4]
MS specialist nurse telephone calls	65	0.86 (1.26) [0-6]	62	0.47 (0.99) [0-6]
Physiotherapist contacts (home)	65	3.20 (11.52) [0-73]	62	1.81 (5.60) [0-33]
Occupational therapist contacts (home)	65	1.15 (1.83) [0-8]	62	1.39 (1.81) [0-6]
Practice nurse contacts (surgery)	65	1.86 (3.69) [0-26]	62	1.40 (2.38) [0-15]
Community nurse contacts (home)	65	2.52 (6.30) [0-36]	62	2.32 (6.55) [0-34]
Chiropodist/Podiatrist contacts (home)	65	0.26 (0.73) [0-3]	62	0.10 (0.47) [0-3]
Chiropodist/Podiatrist contacts (surgery)	65	0.37 (1.40) [0-9]	62	0.15 (0.60) [0-4]
Continence advisor contacts (home)	65	0.06 (0.24) [0-1]	62	0.16 (0.45) [0-2]
Community psychiatric nurse contacts (home)	65	0	62	0
Counsellor contacts	65	0.18 (1.06) [0-7]	62	0.11 (0.77) [0-6]
**Secondary care**				
Hospital stays (nights)	65	2.31 (8.80) [0-45]	62	2.03 (6.57) [0-35]
Visits to A&E	65	0.26 (0.64) [0-4]	62	0.24 (0.69) [0-4]
Days in hospital	65	0.26 (1.19) [0-9]	62	0.23 (1.21) [0-9]
Rehabilitation unit stays (days)	65	0.92 (5.94) [0-46]	62	1.81 (14.22) [0-112]
Neurologist appointments	65	0.62 (0.76) [0-3]	62	0.69 (0.69) [0-2]
MS specialist nurse	65	0.42 (1.04) [0-6]	62	0.76 (1.76) [0-9]

Occupational therapist	65	0.34 (2.03) [0-16]	62	0.16 (0.68) [0-4]
Physiotherapist	65	1.11 (4.23) [0-22]	62	2.58 (5.57) [0-28]
Opthalmologist	65	0.20 (0.59) [0-3]	62	0.26 (0.87) [0-5]
Orthotist	65	0.11 (0.40) [0-2]	62	0.16 (0.55) [0-3]
Chiropodist	65	0.06 (0.29) [0-2]	62	0.14 (0.47) [0-2]
Speech therapist	65	0.02 (0.12) [0-1]	62	0.11 (0.77) [0-6]
Continence advisor	65	0.17 (0.55) [0-3]	62	0.27 (0.63) [0-3]
Psychologist	65	0.06 (0.30) [0-2]	62	
Psychiatrist	65	0	62	0
Pain clinic	65	0.14 (0.58) [0-3]	62	0.08 (0.33) [0-2]
Urologist	65	0	62	0.16 (0.41) [0-2]
New wheelchair	65	0.23 (0.46) [0-2]	62	0.27 (0.52) [0-2]
Medication use (weeks of use):				
Disease-modifying medicine	65	1.14 (5.11) [0-36]	62	2.35 (8.01) [0-36]
Botulinum toxin injections	65	0	62	0.15 (0.44) [0-2]
Phenol injections	65	0	62	0.02 (0.13) [0-1]
Intravenous steroids	65	0	62	0
Steroid tablets	65	0.05 (0.21) [0-1]	62	0.06 (0.25) [0-1]
**Social care**				
Home care worker contacts	65	40.08 (152.28) [0-1120]	62	102.55 (370.95) [0-2688]
Social worker/Care manager contacts	65	0.23 (0.61) [0-3]	62	0.71 (2.26) [0-17]
Respite unit/facility stays (day)	65	0.54 (3.11) [0-21]	62	0
Day care centre (days)	65	1.29 (5.91) [0-41]	62	2.31 (8.89) [0-40]
**Informal care**				
Friends/relatives days off work to help	65	0.54 (2.05) [0-12]	62	0.85 (2.59) [0-17]
Friends/relatives help with (total hours):				
Personal care	65	200.62 (260.08) [0-1,224]	62	229.10 (253.98) [0-1,120]

DIY/home maintenance	65	55.33 (72.79) [0-340]	62	68.95 (102.33) [0-504]
Housework/laundry	65	202.72 (191.19) [0-868]	62	236.65 (256.73) [0-1,456]
Getting out of house/providing transport	65	138.07 (143.40) [0-640]	62	204.93 (224.70) [0-1,000]
Preparing meals	65	311.78 (210.64) [0-980]	62	314.73 (235.66) [0-896]
Gardening	65	88.97 (111.48) [0-436]	62	106.62 (153.08) [0-784]
Shopping	65	89.46 (99.59) [0-700]	62	98.35 (104.33) [0-672]
Taking care of pets	65	72.69 (125.73) [0-536]	62	84.89 (144.79) [0-556]
**Own expenses**				
Paid for help with tasks, equipment, adaptations, therapy	65	49 (75.4%)	63	54 (85.7%)
Cost	**n**	**Mean (SD) [range] (£, 2016)**	**n**	**Mean (SD) [range] (£, 2016)**
**Primary care**				
GP contacts (surgery)	65	66.46 (101.37) [0-540]	62	57.05 (81.09) [0-513]
GP contacts (home)	65	17.89 (47.26) [0-308]	62	13.79 (42.66) [0-274]
GP telephone calls	65	43.26 (53.51) [0-213]	62	35.73 (47.04) [0-256]
MS specialist nurse contacts (home)	65	30.34 (56.42) [0-204]	62	18.65 (52.48) [0-272]
MS specialist nurse telephone calls	65	28.43 (41.61) [0-198]	62	15.44 (32.57) [0-198]
Physiotherapist contacts (home)	65	169.60 (610.81) [0-3,869]	62	95.74 (297.01) [0-1,749]
Occupational therapist contacts (home)	65	88.85 (140.95) [0-616]	62	106.81 (139.67) [0-462]
Practice nurse contacts (surgery)	65	17.31 (34.28) [0-242]	62	13.05 (22.11) [0-140]
Community nurse contacts (home)	65	93.35 (233.01) [0-1,332]	62	85.94 (242.19) [0-1,258]
Chiropodist/Podiatrist contacts (home)	65	10.72 (30.13) [0-123]	62	3.97 (19.23) [0-123]
Chiropodist/Podiatrist contacts (surgery)	65	15.14 (57.33) [0-369]	62	5.95 (24.45) [0-164]
Continence advisor contacts (home)	65	5.11 (20.10) [0-83]	62	13.39 (37.40) [0-166]
Community psychiatric nurse contacts (home)	65	0	62	0

Counsellor contacts	65	8.12 (46.60) [0-308]	62	4.97 (33.90) [0-264]
Sub-total	*65*	*594.58 (831.29) [0-4,946]*	*62*	*470.46 (681.94) [0-3,900]*
**Secondary care**				
Hospital stays (nights)	65	1,050.39 (4,004.56) [0-20,483]	62	925.03 (2,988.69) [0-15,931]
Visits to A&E	65	38.66 (95.20) [0-591]	62	35.76 (102.56) [0-591]
Days in hospital	65	96.51 (438.92) [0-3,321]	62	83.32 (445.31) [0-3,321]
Rehabilitation unit stays (days)	65	91.14 (586.26) [0-4542]	62	178.35 (1404.34) [0-11058]
Neurologist appointments	65	103.08 (128.02) [0-503]	62	116.17 (115.84) [0-335]
MS specialist nurse	65	18.28 (45.95) [0-264]	62	33.35 (77.53) [0-396]
Occupational therapist	65	21.97 (131.63) [0-1,040]	62	10.48 (44.33) [0-260]
Physiotherapist	65	54.07 (206.57) [0-1,074]	62	125.96 (272.06) [0-1,367]
Opthalmologist	65	11.20 (33.12) [0-168]	62	14.45 (48.55) [0-280]
Orthotist	65	12.82 (47.64) [0-238]	62	19.20 (65.37) [0-357]
Chiropodist	65	7.18 (26.37) [0-140]	62	14.30 (38.32) [0-187]
Speech therapist	65	1.48 (11.97) [0-97]	62	10.90 (74.36) [0-579]
Continence advisor	65	14.05 (45.39) [0-249]	62	22.76 (52.43) [0-249]
Psychologist	65	10.38 (50.57) [0-337]	62	0
Psychiatrist	65	0	62	0
Pain clinic	65	19.28 (81.17) [0-418]	62	11.23 (45.79) [0-278]
Urologist	65	0	62	17.44 (44.49) [0-219]
New wheelchair	65	44.08 (87.85) [0-382]	62	52.37 (98.85) [0-382]
Medication use (weeks of use):				
Disease-modifying medicine	65	186.14 (835.63) [0-5,886]	62	385.02 (1,309.71) [0-5,886]
Botulinum toxin injections	65	0	62	6.69 (20.17) [0-92]
Phenol injections	65	0	62	0.08 (0.62) [0-5]
Intravenous steroids	65	0	62	0
Steroid tablets	65	6.69 (30.66) [0-145]	62	9.35 (35.91) [0-145]

Sub-total	*65*	*1,787.40 (4,155.02) [0-20,946]*	*62*	*2,074.17 (3,836.70) [0-20,663]*
**Social care**				
Home care worker contacts	65	276.53 (1050.71) [0-7,728]	62	707.58 (2559.54) [0-18,547]
Social worker/Care manager contacts	65	12.69 (33.36) [0-165]	62	39.03 (124.14) [0-935]
Respite unit/facility stays (day)	65	75.92 (438.21) [0-2,961]	62	0
Day care centre (days)	65	112.43 (514.41) [0-3,567]	62	200.66 (773.61) [0-3,480]
Sub-total	*65*	*477.58 (1,359.09) [0-7,728]*	*62*	*947.28 (3,086.93) [0-22,614]*
Total cost to NHS/PSS	*65*	*2,859.56 (4,958.43) [27-28,017]*	*62*	*3,491.91 (5,408.15) [94-27,730]*
**Informal care**				
Friends/relatives days off work to help	65	55.69 (212.49) [0-1,241]	62	87.58 (267.74) [0-1,758]
Friends/relatives help with (total hours):				
Personal care	65	2,766.49 (3,586.48) [0-16,879]	62	3,159.33 (3,502.34) [0-15,445]
DIY/home maintenance	65	762.99 (1,003.81) [0-4,689]	62	950.87 (1,411.16) [0-6,950]
Housework/laundry	65	2,795.54 (2,636.48) [0-11,970]	62	3,263.34 (3,540.35) [0-20,078]
Getting out of house/providing transport	65	1,904.00 (1,977.47) [0-8,826]	62	2,826.02 (3,098.60) [0-13,790]
Preparing meals	65	4,299.51 (2,904.74) [0-13,514]	62	4,340.07 (3,249.69) [0-12,356]
Gardening	65	1,226.89 (1,537.30) [0-6,012]	62	1,470.28 (2,110.97) [0-10,811]
Shopping	65	1,233.63 (1,373.30) [0-9,653]	62	1,356.27 (1,438.69) [0-9,267]
Taking care of pets	65	1,002.43 (1,733.82) [0-7,391]	62	1,170.59 (1,996.64) [0-7,667]
Sub-total	*65*	*16,047.16 (9,944.57) [0-37,950]*	*62*	*18,624.35 (13,589.22) [0-55,436]*
**Own expenses**				
Personal care		403.85 (1,368.30) [0-7,530]		164.65 (763.73) [0-5,240]
Domestic care		583.92 (1,330.11) [0-8,600]		487.77 (741.93) [0-3,320]
Adaptations to home		574.31 (3,570.69) [0-28,000]		712.50 (2,281.82) [0-13,000]

Special equipment		101.22 (260.29) [0-1,250]		393.69 (1,328.49) [0-8,185]
Private physiotherapy		30.26 (105.49) [0-600]		49.89 (130.06) [0-560]
Private counselling		0.86 (5.31) [0-40]		0
Other		1,209.36 (5,604.76) [0-40,527]		286.07 (631.95) [0-3,570]
Sub-total	*65*	*2,999.25 (6,951.45) [0-40,527]*	*62*	*2,117.50 (3,437.69) [0-15,275]*
Total cost to NHS, PSS, patients and carers	***65***	***21,905.97 (12,147.65) [555-55,517]***	***62***	***24,233.75 (13,464.93) [2,045-58,469]***
Cost at baseline	**n**	**Mean (SD) [range] (£, 2016)**	**n**	**Mean (SD) [range] (£, 2016)**
**Primary care**				
GP contacts (surgery)	71	55.52 (89.54) [0-540]	69	45.39 (60.11) [0-270]
GP contacts (home)	71	11.08 (30.47) [0-205]	69	8.92 (26.65) [0-171]
GP telephone calls	71	36.6 (63.00) [0-426]	69	23.15 (30.94) [0-128]
MS specialist nurse contacts (home)	71	23.94 (39.98) [0-136]	69	19.71 (38.86) [0-136]
MS specialist nurse telephone calls	71	42.30 (113.66) [0-858]	69	17.22 (28.07) [0-99]
Physiotherapist contacts (home)	71	144.07 (530.01) [0-4,134]	69	79.12 (228.26) [0-1,272]
Occupational therapist contacts (home)	71	69.68 (117.65) [0-539]	69	90.39 (164.38) [0-770]
Practice nurse contacts (surgery)	70	15.01 (35.02) [0-242]	69	12.80 (46.28) [0-372]
Community nurse contacts (home)	71	54.20 (146.78) [0-888]	69	35.39 (128.71) [0-962]
Chiropodist/Podiatrist contacts (home)	71	10.39 (40.83) [0-246]	69	2.97 (16.22) [0-123]
Chiropodist/Podiatrist contacts (surgery)	71	4.62 (29.85) [0-246]	69	7.13 (32.95) [0-246]
Continence advisor contacts (home)	71	14.03 (64.27) [0-498]	69	4.81 (24.17) [0-166]
Community psychiatric nurse contacts (home)	71	0.51 (4.27) [0-36]	69	0
Counsellor contacts	71	8.68 (45.62) [0-352]	69	0
Sub-total	*70*	*497.42 (643.21) [0-4,479]*	*69*	*347.01 (419.92) [0-2,306]*

**Secondary care**				
Hospital stays (nights)	71	7,50.07 (3,602.22) [0-29,586]	69	9,49.92 (5,293.05) [0-40,965]
Visits to A&E	71	29.14 (81.41) [0-443]	69	29.99 (82.45) [0-443]
Days in hospital	71	67.56 (319.93) [0-2,214]	69	5.35 (44.42) [0-369]
Rehabilitation unit stays (days)	71	90.39 (761.61) [0-6,417]	69	11.45 (95.09) [0-790]
Neurologist appointments	71	82.57 (88.96) [0-335]	69	87.39 (97.87) [0-335]
MS specialist nurse	71	17.35 (36.80) [0-264]	69	18.49 (39.50) [0-264]
Occupational therapist	71	16.48 (61.87) [0-390]	69	10.36 (54.12) [0-390]
Physiotherapist	71	77.68 (213.33) [0-1,171]	69	55.18 (129.93) [0-732]
Opthalmologist	71	10.25 (31.83) [0-224]	69	8.11 (25.90) [0-168]
Orthotist	71	13.41 (37.92) [0-119]	69	12.08 (46.32) [0-238]
Chiropodist	71	11.82 (45.09) [0-280]	69	0
Speech therapist	71	1.36 (11.45) [0-97]	69	2.80 (16.31) [0-97]
Continence advisor	71	14.03 (34.32) [0-166]	69	22.86 (46.96) [0-166]
Psychologist	71	61.76 (481.46) [0-4,048]	69	4.89 (28.50) [0-169]
Psychiatrist	71	6.00 (50.56) [0-426]	69	0
Pain clinic	71	13.73 (53.43) [0-278]	69	26.23 (117.65) [0-835]
Urologist	71	1.54 (12.98) [0-109]	69	6.34 (25.75) [0-109]
New wheelchair	71	24.21 (64.00) [0-191]	69	44.29 (81.20) [0-191]
Medication use (weeks of use):				
Disease-modifying medicine	71	133.56 (589.06) [0-4,251]	69	281.98 (975.33) [0-4,251]
Botulinum toxin injections	71	1.30 (7.68) [0-46]	69	5.34 (16.83) [0-92]
Phenol injections	71	0	69	0
Intravenous steroids	71	0	69	0.84 (6.98) [0-58]
Steroid tablets	71	6.13 (29.38) [0-145]	69	10.51 (45.30) [0-290]
Sub-total		*1,430.35 (4,462.07) [0-36,390]*		*1,594.39 (5,313.42) [0-40,965]*
**Social care**				

Home care worker contacts	71	110.50 (344.64) [0-1,656]	69	281.40 (709.59) [0-3,767]
Social worker/Care manager contacts	71	15.49 (37.40) [0-165]	69	19.13 (58.01) [0-330]
Respite unit/facility stays (day)	71	47.66 (354.26) [0-2,961]	69	61.30 (509.23) [0-4,230]
Day care centre (days)	71	52.69 (313.85) [0-2,088]	69	223.17 (858.08) [0-4,524]
Sub-total	*71*	*226.34 (696.31) [0-4,669]*	*69*	*585.01 (1,420.03) [0-7,542]*
Total cost to NHS/PSS	***70***	***2,177.78 (4,800.87) [27-38,409]***	***69***	***2,526.41 (5,648.44) [9-42,778]***
**Informal care**				
Friends/relatives days off work to help	71	152.95 (661.98) [0-4,964]	69	121.41 (490.89) [0-3,723]
Friends/relatives help with (total hours):				
Personal care	71	2,139.21 (2,974.91) [0-15,224]	69	2,111.67 (2,816.62) [0-13,569]
DIY/home maintenance	71	585.61 (769.43) [0-3,310]	69	614.43(1,108.30) [0-5,957]
Housework/laundry	71	2,893.57 (2,665.35) [0-9,929]	69	2,162.27 (2,253.45) [0-8,274]
Getting out of house/providing transport	71	1,670.88 (1,652.62) [0-6,950]	69	1,760.32 (2,089.37) [0-13,900]
Preparing meals	71	3,059.05 (2,117.10) [0-9,267]	69	2,587.72 (2,247.05) [0-9,267]
Gardening	71	1,051.38 (1,596.86) [0-9,267]	69	746.10 (996.41) [0-4,633]
Shopping	71	843.71 (680.08) [0-2,648]	69	737.94 (702.28) [0-2,648]
Taking care of pets	71	564.03 (1,119.05) [0-4,633]	69	557.60 (1,113.30) [0-4,633]
Sub-total	*71*	*12,960.41 (8,429.73) [0-35,413]*	*69*	*11,399.47 (9,738.67) [0-46,665]*
**Own expenses**				
Personal care	71	170.43 (945.41) [0-7,140]	69	140.70 (774.41) [0-6,000]
Domestic care	71	350.25 (725.01) [0-3,900]	69	383.07 (605.73) [0-2,700]
Adaptations to home	71	552.68 (3,585.20) [0-30,000]	69	1,122.90 (4,397.07) [0-32,000]
Special equipment	71	574.97 (2,584.40) [0-20,000]	69	224.41 (960.49) [0-7,500]
Private physiotherapy	71	21.16 (80.32) [0-420]	69	47.29 (165.57) [0-1,200]
Private counselling	71	0.56 (4.75) [0-40]	69	0

Other	71	750.44 (3,107.21) [0-22,500]	69	146.15 (346.91) [0-2,060]
Sub-total	*71*	*2,420.50 (5,443.01) [0-30,000]*	*69*	*2,064.51 (4,675.64) [0-32,500]*
Total cost to NHS, PSS, patients and carers	***70***	***17,649.87 (11,596.99) [387-69,782]***	***69***	***15,990.39 (11,812.41) [703-49,732]***

Table A7: Incremental cost-effectiveness analyses and sensitivity analyses

Analysis	*Difference, adjusted for baseline and participant covariates* Mean (95% CI)*	*Incremental cost-effectiveness ratio (ICER), cost (£) per QALY*
**Base case**		
Total cost to NHS and personal social services	£268.47 (-£1,093.79, £2,051.38)	£14,733
EQ-5D: QALYs (36 weeks)	0.018 (-0.014, 0.051)	
**Sensitivity analyses**		
1. Societal perspective		
Cost to NHS, PSS, patients and informal carers	-£2,192.41 (-£5,755.23, £1,163.43)	Standing frame intervention is dominant
EQ-5D: QALYs (36 weeks)	0.018 (-0.016 to 0.050)	
2. Adjustment to intervention cost based on re-use of 30% of standing frames over 10 years		
Cost to NHS and PSS	-£174.96 (-£1,524.95, £1,560.19)	Standing frame intervention is dominant
EQ-5D: QALYs (36 weeks)	0.018 (-0.014, 0.051)	
3. Adjustment to intervention cost based on re-use of 30% of standing frames over 5 years		
Cost to NHS and PSS	-£123.92 (-£1,473.91, £1,611.23)	Standing frame intervention is dominant
EQ-5D: QALYs (36 weeks)	0.018 (-0.014, 0.051)	
4. Higher cost for intervention booklet and DVD (£10)		
Cost to NHS and PSS	£276.47 (-£1,230.67, £1,994.11)	£15,172
EQ-5D: QALYs (36 weeks)	0.018 (-0.016 to 0.050)	

*Value at baseline, EDSS category (>=7.5,<7.5) and region.

Figure A2: The cost-effectiveness plane of bootstrapped replicates of incremental costs and incremental QALYs

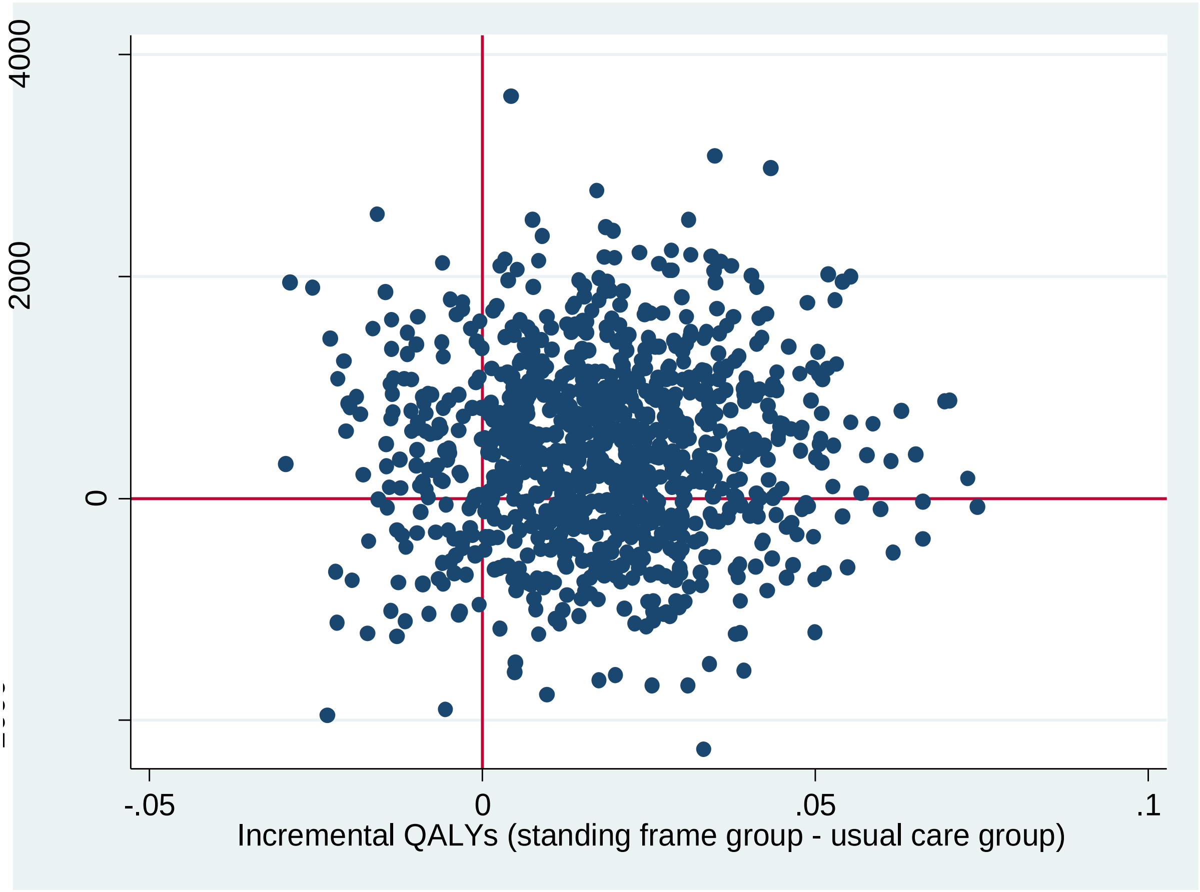

Supplement: Supplementary appendix [file mmc1.pdf]
